# Butyrolactone I blocks the transition of acute kidney injury to chronic kidney disease in mice by targeting JAK1

**DOI:** 10.1002/mco2.70064

**Published:** 2025-01-21

**Authors:** Zijun Zhang, Ziming Zhao, Changxing Qi, Xiaotian Zhang, Yang Xiao, Chengjuan Chen, Yu Zou, Xia Chen, Lianghu Gu, Jianzheng Huang, Kun Huang, Ming Xiang, Tiantai Zhang, Qingyi Tong, Yonghui Zhang

**Affiliations:** ^1^ Hubei Key Laboratory of Natural Medicinal Chemistry and Resource Evaluation School of Pharmacy Tongji Medical College Huazhong University of Science and Technology Wuhan China; ^2^ State Key Laboratory of Bioactive Substances and Function of Natural Medicine Institute of Materia Medica Chinese Academy of Medical Sciences Peking Union Medical College Beijing China; ^3^ Institute of Pharmaceutical Process Hubei Province Key Laboratory of Occupational Hazard Identification and Control School of Medicine Wuhan University of Science and Technology Wuhan China

**Keywords:** AKI–CKD transition, butyrolactone I, ferroptosis, JAK1

## Abstract

Chronic kidney disease (CKD) is a disease that affects more than 850 million people. Acute kidney injury (AKI) is a common cause of CKD, and blocking the AKI–CKD transition shows promising therapeutic potential. Herein, we found that butyrolactone I (BLI), a natural product, exerts significant nephroprotective effects, including maintenance of kidney function, inhibition of inflammatory response, and prevention of fibrosis, in both folic acid‐ and ureteral obstruction‐induced AKI–CKD transition mouse models. Notably, BLI showed greater blood urea nitrogen reduction and anti‐inflammatory effects than telmisartan. Bioinformatics analysis and target confirmation assays suggested that BLI directly binds to JAK1, and kinase inhibition assay confirmed it is a potent JAK1inhibitor with an IC_50_ of 0.376 µM. Experiments in JAK1‐knockdown mice also proved that BLI targets JAK1 to work. Furthermore, BLI demonstrated nephroprotective effects and safety comparable to ivarmacitinib, the well‐known JAK1 inhibitor. Mechanistically, BLI targets JAK1 and inhibits its phosphorylation and JAK‐STAT activation, subsequently regulating the downstream signaling pathways to inhibit reactive oxygen species production, inflammation, and ferroptosis, thereby preventing the occurrence of kidney fibrosis and blocking the AKI–CKD transition process. This study demonstrates for the first time that BLI is a JAK1 inhibitor and a promising candidate for delaying CKD progression, which warrants further investigation.

## Graphical abstract

In FA‐ or UUO‐induced CKD models, BLI targets JAK1 and inhibits its phosphorylation and activation of the JAK‐STAT signaling pathway, thereby inhibiting multiple downstream events that promote kidney fibrosis.

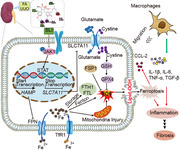

## 1 INTRODUCTION

Chronic kidney disease (CKD) is a chronic progressive destruction of kidney structure and function.^1^, ^2^ Recent data show that the number of CKD patients worldwide is more than 850 million,^3^ with a prevalence of more than 10%,^4^, ^5^ and China has the largest number of CKD patients (132.3 million cases and 9.5% prevalence).^6^ In 2017, CKD was identified as the 12th leading cause of death globally and is projected to be the fifth leading cause of death globally by 2040.^6^, ^7^ Moreover, due to the influence of the aging population, obesity, diabetes, and other factors, the multifaceted burden of CKD (prevalence, morbidity, mortality, and costs) is relentlessly growing annually.^7^ This situation indicates that CKD has become a serious global public health problem, and there is an urgent need to strengthen relevant research and develop effective prevention or treatment strategies.

Acute kidney injury (AKI) is a common clinical syndrome with complex pathogenesis that is characterized by a rapid decline in kidney function in a short period of time and can lead to CKD without timely intervention or incomplete recovery.^8^ Therefore, AKI is one of the greatest risk factors for CKD development and progression. Factors that contribute to the progression of AKI to CKD include nephron loss, oxidative stress, chronic inflammation, ferroptosis, metabolic changes, vascular damage, reduced kidney regenerative capacity, and fibrosis.^5^, ^9^ Consequently, blocking the AKI–CKD transition by inhibiting the signaling pathways associated with the above events is essential for maintaining kidney function and is currently the most promising way to prevent the development of CKD.^9^ However, there is still a lack of research on the development of drugs or effective therapies targeting these mechanisms.

Butyrolactone I (BLI, also known as olomoucine) is a natural product produced by *Aspergillus terreus* isolated from several marine‐derived samples.^10^ It has been reported that BLI has anti‐tumor,^11^ anti‐inflammatory,^12^, ^13^, ^14^, ^15^ and hypoglycemic^16^, ^17^ effects, but the research on the mechanism of action is still insufficient. The reason is that the natural content of BLI is rare, and it is difficult to obtain a large amount of BLI, which is also a common bottleneck in the research and development of many natural products.^18^, ^19^ In this study, a silica gel chromatography column combined with recrystallization was used to overcome the limitation of low yield, and a large amount (556.2 g) of BLI was isolated from the large‐scale fermentation products of *Aspergillus terreus*, which provided the possibility for extensive activity screening and further development.

Moreover, for the first time, we report the nephroprotective effects of BLI on AKI–CKD transition models. According to the latest clinical guidelines, renin–angiotensin system (RAS) inhibitors are the drugs of choice for the treatment of almost all types of CKD and have been in use for decades.^3^ Thus, we selected the classic RAS inhibitor telmisartan as a positive control. Notably, we found that BLI had significantly greater blood urea nitrogen (BUN) reduction and anti‐inflammatory effects than telmisartan in mouse AKI–CKD transition models. We also demonstrated for the first time that BLI exerts a nephroprotective effect by targeting JAK1 to inhibit the JAK‐STAT signaling pathway and its downstream events. Given the large number of successful precedents of natural products in drug development,^19^, ^20^ our study highlights the potential of BLI as a drug candidate for preventing the AKI–CKD transition and CKD progression in patients.

## 2 RESULTS

### 2.1 BLI exerts nephroprotective effects in mouse AKI–CKD transition models

**FIGURE 1:**
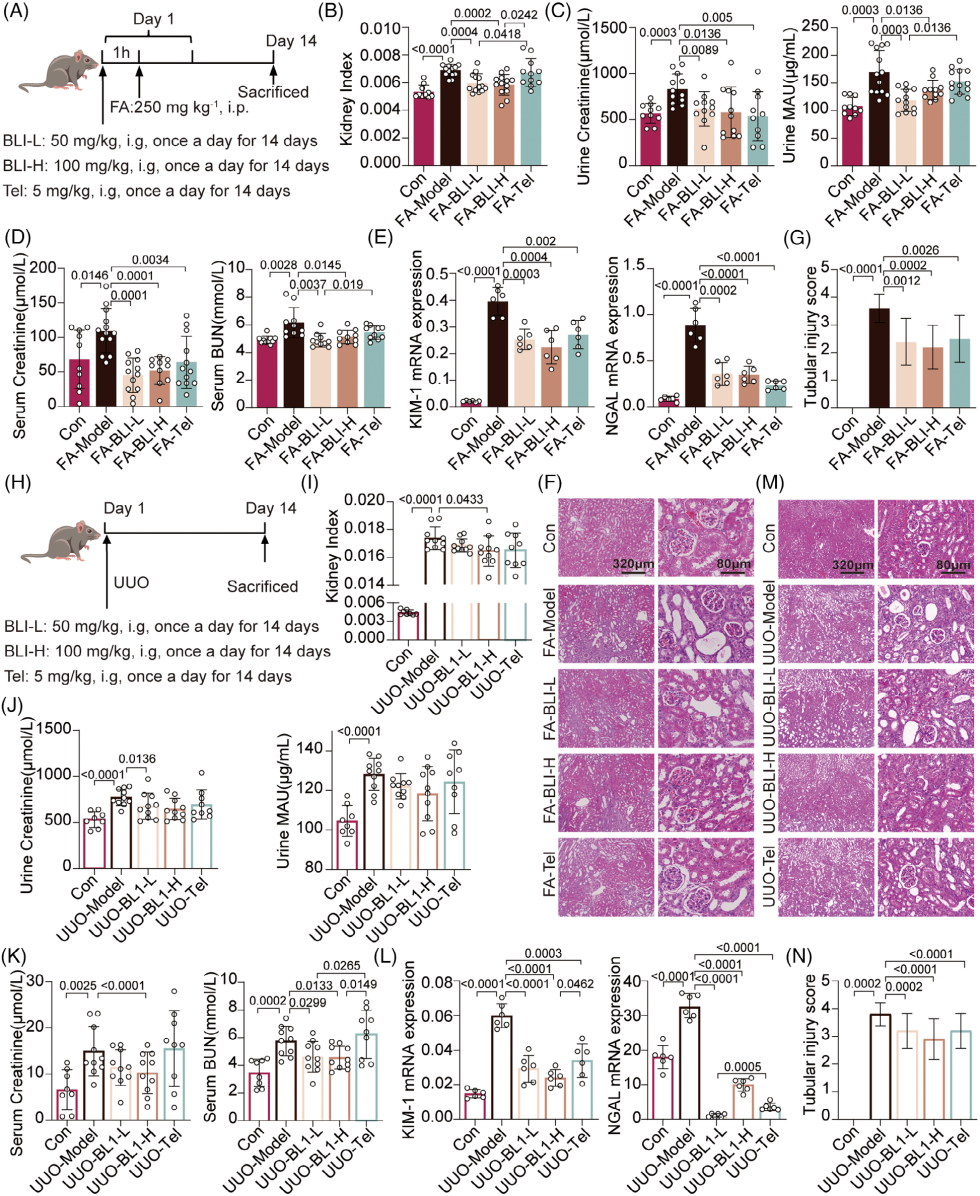
BLI exerts nephroprotective effects on mouse AKI–CKD transition models. The results from the FA‐induced AKI–CKD transition model are shown in (A–F): (A) Schematic of the experiment. (B) Kidney indices of the mice. Kidney index = kidney weight/body weight (*n* ≥10). (C) The levels of urinary Cr and MAU (*n* ≥10). (D) The levels of serum BUN and Cr (*n* ≥10). (E) mRNA levels of several kidney injury biomarkers (*n* = 6). (F) and (G) Representative H&E staining and pathological scores of the kidneys (*n* = 5). The results from the UUO‐induced AKI–CKD transition model are shown in (H–N): (H) Scheme of the experiment. (I) Kidney indices of the mice. (J) The levels of urinary Cr and MAU. (K) The levels of serum Cr and BUN (*n* ≥ 8). (L) mRNA levels of several kidney injury biomarkers (*n* = 6). (M) and (N) Representative H&E staining and pathological scores of the kidneys (*n* = 5). Significant *p* values are indicated on figure panels. Scale bars were as shown in the figure.

Folic acid (FA)‐induced and unilateral ureteral obstruction (UUO) kidney injury mouse models are widely used in the study of AKI–CKD transition or CKD.^21^ We first investigated the effect of BLI on the FA‐induced model (Figure 1A). FA significantly increased the mouse kidney indices,^22^ whereas these changes were reversed by BLI (Figure 1B). In clinical settings, the most commonly utilized parameters for detecting kidney injury include blood urea nitrogen (BUN), creatinine (Cr), and microalbuminuria (MAU) levels.^23^ We found that FA challenge significantly increased the serum BUN, Cr, urinary Cr, and MAU levels in mice, while the serum and urinary Cr and urinary MAU levels in mice treated with BLI did not increase (Figure 1C,D). We subsequently investigated the mRNA expression of kidney injury molecule‐1 (*KIM‐1*) and neutrophil gelatinase‐associated lipocalin (*NGAL*), two known tubule injury biomarkers for early kidney injury.^24^ Treatment with BLI significantly attenuated the increase in *KIM‐1* and *NGAL* levels induced by FA (Figure 1E). Moreover, hematoxylin and eosin (H&E) staining of the kidneys also demonstrated the protective effects of BLI on kidney damage. Mice in the FA‐induced model exhibited severe tubular dilatation, obvious tubular epithelial cell edema, and significant cast formation, which were not observed in the BLI treatment group (Figure 1F,G).

To further confirm these findings, we conducted studies using the UUO model (Figure 1H). Consistently, BLI had protective effects against functional kidney injury (Figure 1I–L) and structural organ damage (Figure 1M,N) in mice. Taken together, these data demonstrated that BLI exerts nephroprotective effects on AKI–CKD transition models. As the cornerstone of kidney‐preserving pharmacologic therapy in the clinic is RAS blockade, we used telmisartan as a positive control.^3^ Notably, in terms of multiple kidney function indicators, the nephroprotective effect of BLI was no worse than or even better than that of the positive control telmisartan (Tel). Especially for the reduction in serum BUN, the effect of BLI was significantly greater than that of telmisartan in both models; in the FA model, the advantages of BLI over telmisartan were also reflected in the improvements in the kidney index and MAU.

### 2.2 BLI inhibits the inflammatory response in mouse AKI–CKD transition models

**FIGURE 2:**
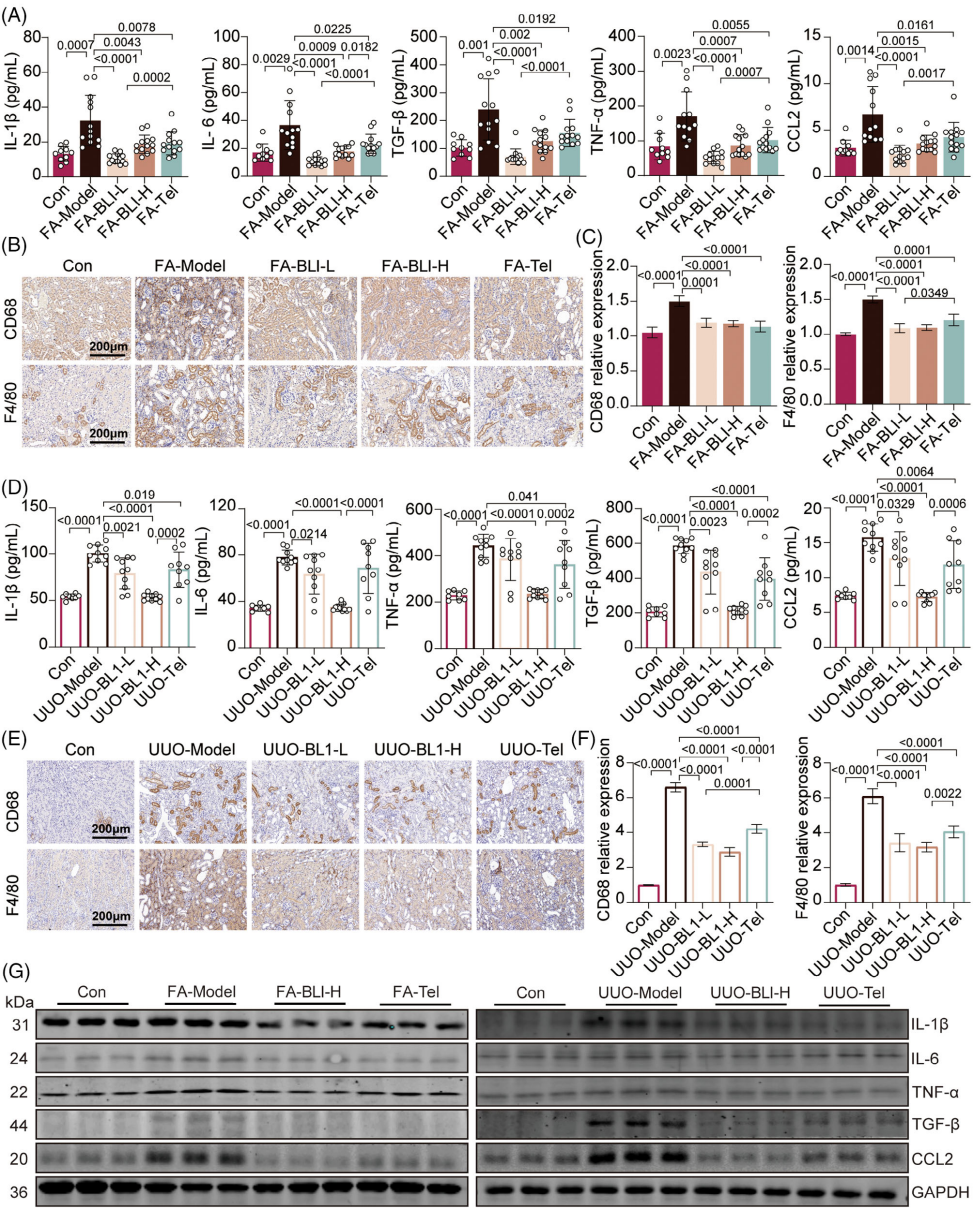
BLI inhibits the inflammatory response in mouse AKI–CKD transition models. The results from the FA‐induced AKI–CKD transition model are shown in (A–C): (A) Serum levels of multiple chemokines (*n* ≥ 10). (B) and (C) Results of immunohistochemical staining for CD68 and F4/80 in the kidney (*n* = 5). The results from the UUO‐induced AKI–CKD transition model are shown in (D‐F): (D) Serum levels of multiple chemokines (*n* ≥ 8). (E) and (F) Immunohistochemical staining for CD68 and F4/80 in the kidney (*n* = 5). (G) Immunoblotting results of chemokine proteins in mouse kidneys (*n* = 3). Significant *p* values are indicated on figure panels. Scale bars were as shown in the figure.

A dramatic increase in inflammatory cytokine levels is one of the major clinical features of both AKI and CKD, and low‐grade systemic inflammation is also a substantial contributor to the AKI–CKD transition.^25^ We subsequently examined the serum and mRNA expression levels of proinflammatory cytokines in kidney tissues. The levels of interleukin‐1 beta (IL‐1β), interleukin‐6 (IL‐6), transforming growth factor‐beta (TGF‐β), tumor necrosis factor‐alpha (TNF‐α), and C‐C motif chemokine ligand 2 (CCL2) were significantly increased in the FA‐ or UUO‐treated mice; however, these increases were not observed in the BLI‐treated mice (Figure 2A,D). Consistently, BLI significantly inhibited the upregulation of IL‐1β, IL‐6, TGF‐β, TNF‐α, and CCL2 in FA or UUO‐induced kidney tissue, both at the transcriptional and translational levels (Figure 2G and Figure S4). Macrophages have been shown to account for the majority of inflammatory infiltrates in various kidney diseases, including AKI and CKD.^26^ We next investigated the kidney infiltration of macrophages by immunohistochemical staining for CD68 and F4/80. Compared with those in control or BLI‐treated mice, the number of CD68‐ or F4/80‐positive cells significantly increased in mice after FA treatment or UUO surgery (Figure 2B,C,E,F), indicating that BLI treatment markedly decreased the kidney infiltration of macrophages. These results suggest that BLI substantially suppressed the inflammatory response in AKI–CKD transition models. Notably, BLI demonstrated significantly stronger anti‐inflammatory effects than telmisartan in both models.

### 2.3 BLI blocks kidney fibrosis in mouse AKI–CKD transition models

**FIGURE 3:**
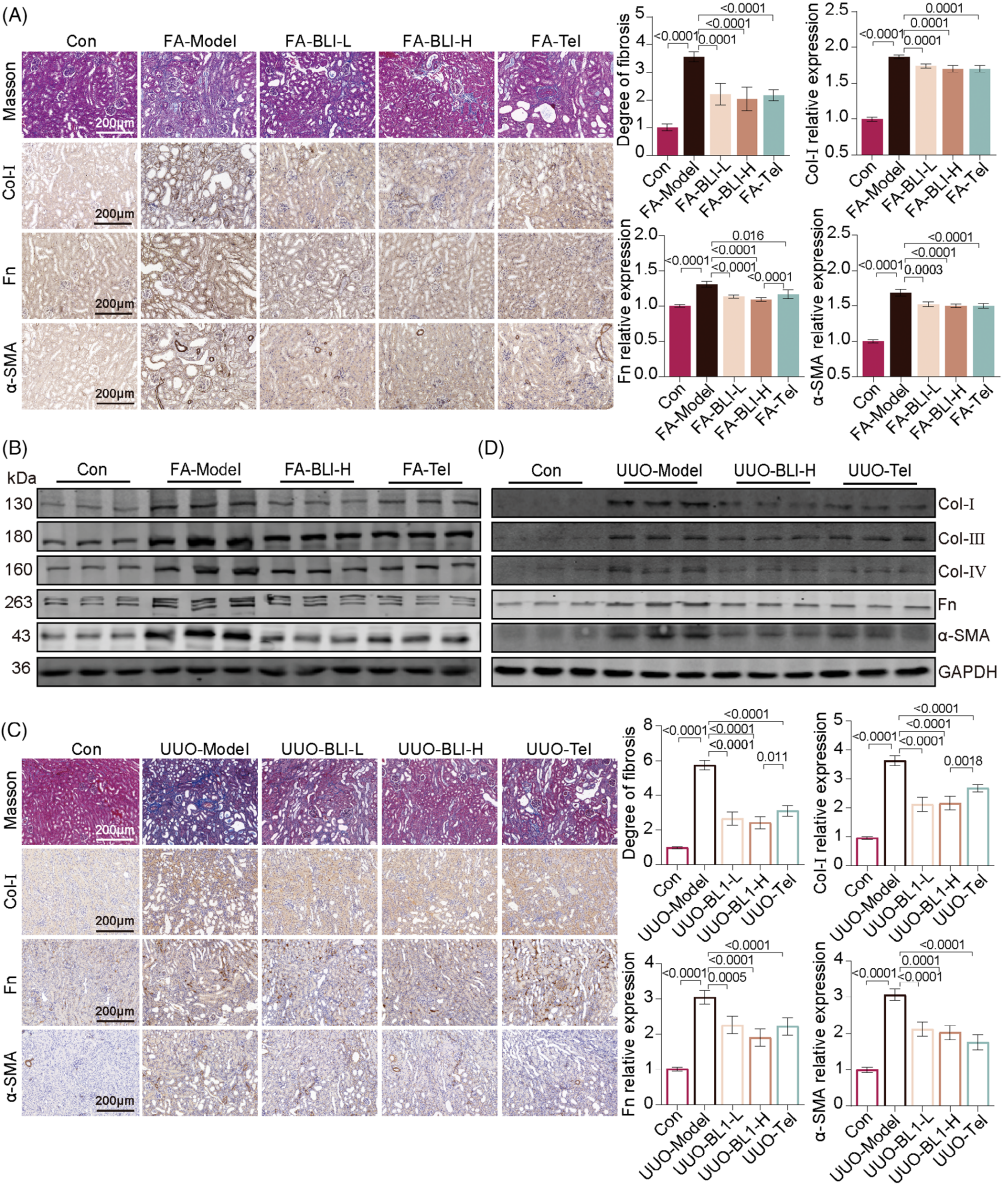
BLI blocks kidney fibrosis in mouse AKI–CKD transition models. (A) and (C) Representative images of Masson or immunohistochemical staining and quantification of fibrosis, Col‐I, Fn, and α‐SMA in kidney sections (*n* = 5). (B) and D) Immunoblotting results of pro‐fibrotic proteins in mouse kidneys (*n* = 3). Significant *p* values are indicated on figure panels. Scale bars were as shown in the figure.

Without treatment, AKI will lead to the continued deterioration of kidney function, and the kidney is in a long‐term proinflammatory, profibrotic state and gradually becomes a fibrotic kidney,^27^, ^28^ which is characterized primarily by the accumulation of extracellular matrix (ECM) components. To evaluate the effectiveness of BLI on kidney fibrosis, the levels of ECM and fibrosis markers were examined. The results from Masson's trichrome staining of the kidneys demonstrated that BLI mitigated ECM accumulation in both AKI–CKD transition models (Figure 3A,C). Collagen I (Col‐I), collagen III (Col‐III), collagen IV (Col‐IV), fibronectin (Fn), and α‐smooth muscle actin (α‐SMA) are the main components of the ECM. Then, immunohistochemical staining and immunoblotting analysis were employed to assess the expression of this protein. The results showed that the expressions of profibrotic proteins such as Col‐I, Col‐III, Col‐IV, Fn, and α‐SMA were significantly increased in mice after FA challenge or UUO surgery, while these changes were not observed in the BLI‐treated mice (Figure 3B,D). These results suggested that BLI blocks the AKI–CKD transition by inhibiting kidney fibrosis.

### 2.4 BLI inhibits kidney ferroptosis in both mouse and cell models

**FIGURE 4:**
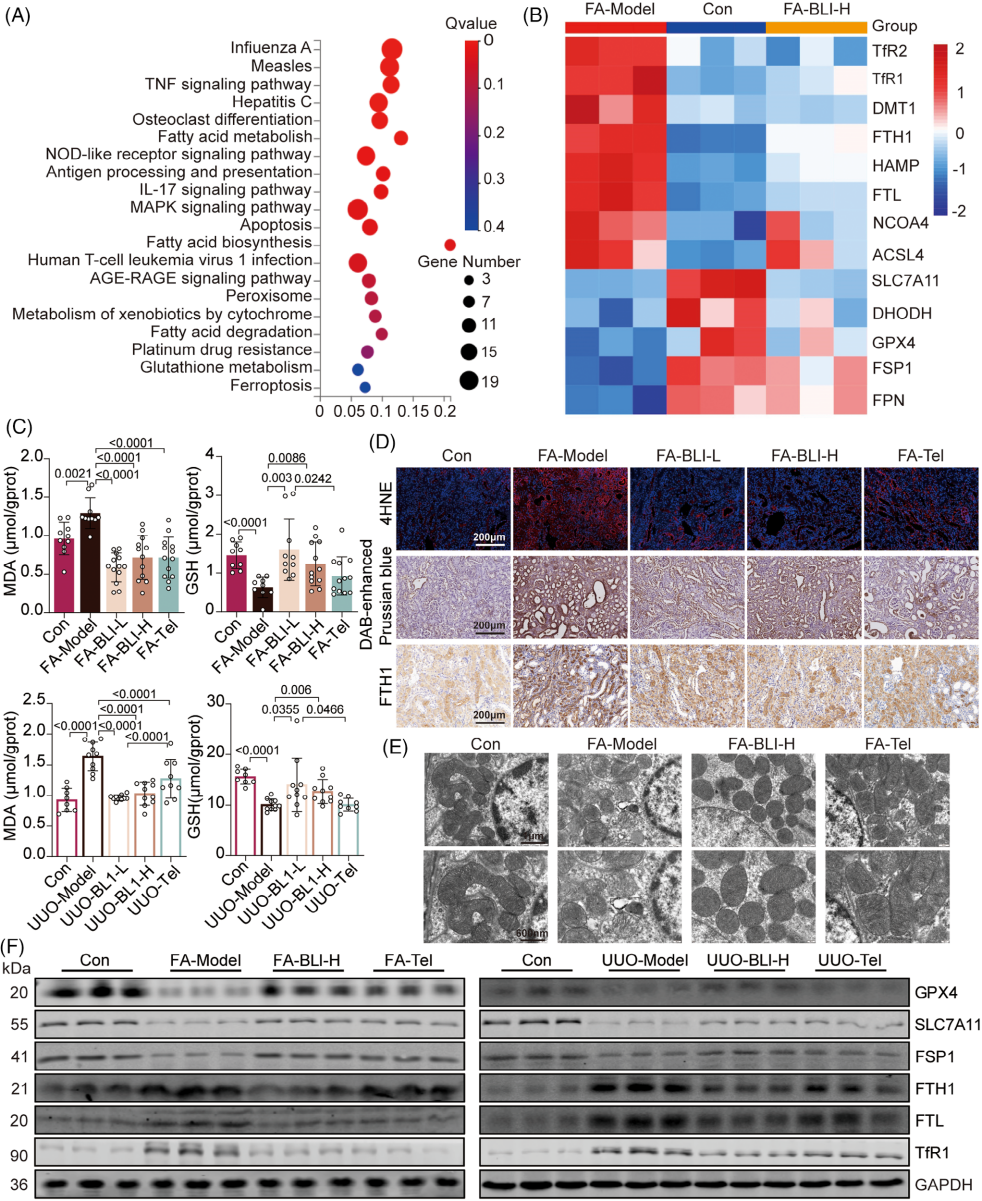
BLI inhibits kidney ferroptosis. RNA sequencing analysis were conducted using the kidney tissues from the FA‐induced mouse model as indicated in Figure 1A. (A) Results of KEGG analysis of the differentially expressed genes from RNA‐seq (*n* = 3). (B) Results of RT‐qPCR (*n* = 3). (C) MDA and GSH levels in kidneys (*n* ≥ 8). (D) Representative images of 4‐HNE, Diaminobenzidine (DAB)‐enhanced Prussian blue and FTH1 staining of the kidneys (*n* = 5). (E) Representative images obtained from transmission electron microscopy of kidney tissues in the FA‐induced mouse model. (F) Immunoblotting results of the proteins in mouse kidneys (*n* = 3). Significant *p* values are indicated on figure panels. Scale bars were as shown in the figure.

The above results illustrate the nephroprotective effect of BLI on the whole body or at the kidney level. To explore the mechanism of action of BLI, we conducted RNA sequencing analysis using the kidney tissues from the FA‐induced mouse model as indicated in Figure 1A. The results showed that genes altered in the BLI‐treated group were enriched in pathways relevant to ferroptosis, such as fatty acid metabolism, glutathione (GSH) metabolism, and ferroptosis (Figure 4A). Ferroptosis is a nonapoptotic form of cell death that is driven by substantial iron accumulation and lipid peroxidation.^29^ According to previous reports, ferroptosis is an important driver of FA‐ or UUO‐induced kidney fibrosis.^30^, ^31^ Therefore, we investigated the mRNA expression of critical genes involved in the signaling pathways that regulate ferroptosis. As depicted in Figure 4B, we observed changes in the expression of multiple genes associated with ferroptosis in the kidneys of the FA‐treated mice compared to those of the control group and found that the degree of changes decreased after BLI treatment. Lipid peroxidation, indicated by the accumulation of malondialdehyde (MDA) and 4‐hydroxynonenal (4‐HNE), as well as GSH depletion and iron accumulation, is the main biochemical characteristic of ferroptosis.^32^ We found that the serum MDA concentration increased and the GSH concentration decreased in both AKI–CKD transition models compared with those in normal mice, but the levels were almost unchanged in BLI‐treated mice (Figure 4C). Immunostaining of kidney specimens revealed that 4‐HNE, the levels of iron, and the levels of proteins involved in iron storage, such as ferritin heavy chain‐1 (FTH1) and transferrin receptor‐1 (TfR1),^33^, ^34^ also increased significantly in both models, but these changes were not observed in the BLI treatment group (Figure 4D and Figure S5). In particular, the level of 4‐HNE in the BLI‐treated group was close to that in the normal group and was significantly greater than that in the positive control group. As ferroptosis coincided with morphologic changes,^35^ transmission electron microscopy studies revealed that BLI significantly protect FA‐induced mitochondrial cristae disappearance and outer membrane rupture in mouse kidney (Figure 4E). Moreover, the immunoblotting results also showed that the expressions of proteins negatively associated with ferroptosis, such as glutathione peroxidase 4 (GPX4), solute carrier family 7 member 11 (SLC7A11) and ferroptosis suppressor protein 1 (FSP1), significantly decreased in both models but were basically similar to that in the normal control group in the BLI‐treated group. Consistent with the immunostaining results, the expressions of proteins positively associated with ferroptosis (FTH1, FTL, and TfR1) were significantly increased in both models, but BLI treatment blocked this process (Figure 4F). These results indicate that BLI blocks the AKI–CKD transition by inhibiting kidney ferroptosis in mice.

**FIGURE 5:**
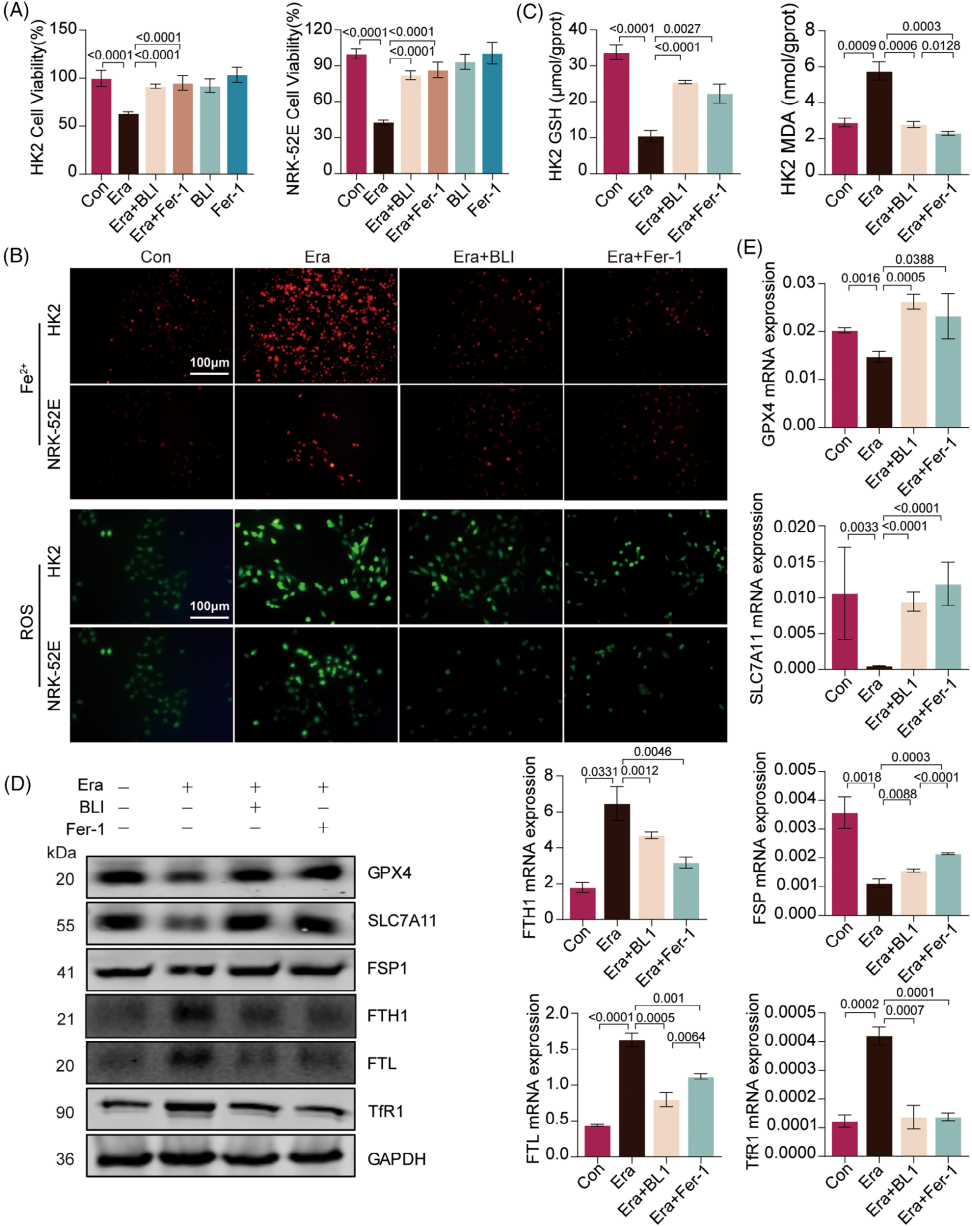
BLI inhibits ferroptosis in HK2 and NRK‐52E cells. (A) Cell viability. The cells were treated for 24 h (Era: 20 µM; BLI: 100 µM; Fer‐1: 2 µM; *n* = 3). (B) Intracellular iron and ROS levels (*n* = 3). (C) MDA and GSH levels in HK2 cells (*n* = 3). (D) Immunoblotting results of the proteins. (E) Results of RT‐qPCR (*n* = 3). Significant *p* values are indicated on figure panels. Scale bars were as shown in the figure.

We also found that, similar to ferrostatin‐1 (Fer‐1), a selective inhibitor of ferroptosis, BLI abolished ferroptosis induced by erastin (Era)^36^ in both NRK‐52E and HK‐2 cells (Figure 5A). Then, we used RhoNox‐1, a fluorescent probe specific for divalent iron ions, to detect changes in the concentration of cellular labile iron. Era treatment significantly increased the fluorescence signals, while BLI or Fer‐1 treatment did not, consistent with the results of the in vivo experiment (Figure 5B and Figure S6A). Free iron can interact with reactive oxygen species (ROS) to form lipid radicals that react with oxygen to induce lipid peroxidation. We next investigated intracellular ROS levels in cells, and the results revealed that Era treatment induced ROS generation, but BLI or Fer‐1 treatment did not (Figure 5B and Figure S6A,B). In addition, Era induced significant GSH downregulation and MDA upregulation in both NRK‐52E and HK‐2 cells, but BLI or Fer‐1 treatment blocked these changes (Figure 5C and Figure S6C). Subsequently, we examined biomarkers of ferroptosis. Both the immunoblotting and real‐time quantitative polymerase chain reaction (RT‐qPCR) results showed that BLI or Fer‐1 treatment significantly reversed the changes in the expression of GPX4, SLC7A11, FSP1, FTH1, FTL, and TfR1 induced by Era (Figure 5D,E). Ferroptosis and ROS production are closely related, and mitochondria are important organelles for ROS production.^37^ We found that exposure of HK2 and NRK‐52E cells to Era disrupted the mitochondrial membrane potential (MMP), as indicated by increased green fluorescence, which indicated depolarization of the mitochondrial membrane. However, the cells treated with BLI or Fer‐1 exhibited a normal MMP (Figure S6D). Taken together, these data indicated that BLI mitigates kidney ferroptosis in both mouse and cell models.

### 2.5 BLI exerts its nephroprotective effect by targeting JAK1

**FIGURE 6:**
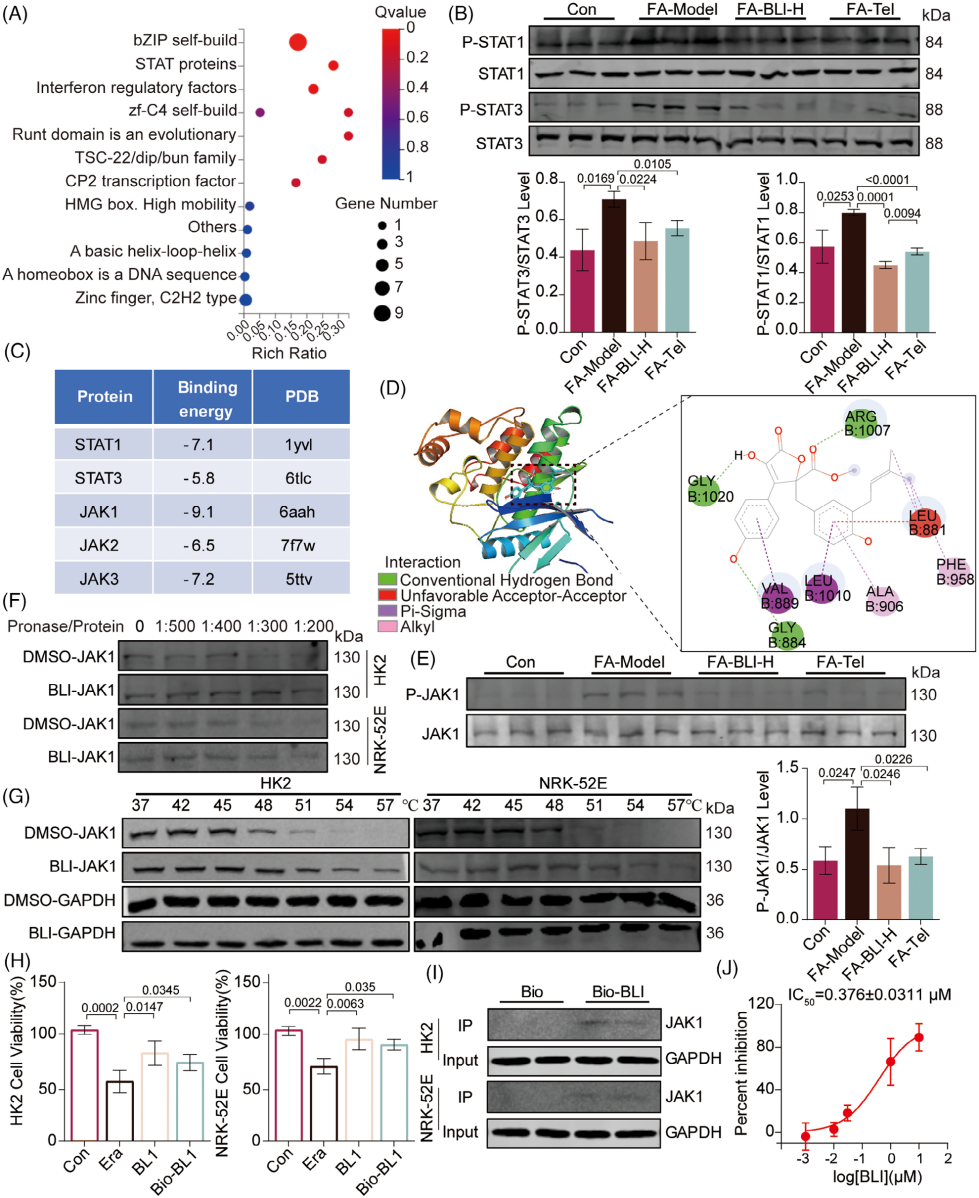
BLI exerts a nephroprotective effect by targeting JAK1. (A) Transcription factor enrichment analysis of the RNA‐seq data (*n* = 3). (B) Results of immunoblotting for p‐STAT1 and p‐STAT3 (*n* = 3). (C) The binding affinities of STAT1, STAT3, JAK1, JAK2, and JAK3, for BLI were assessed by molecular dynamics simulation. (D) 3D and 2D images revealing the binding mode of BLI with JAK1. (E) Immunoblotting results for p‐JAK1 (*n* = 3). (F) Results of CETSA showing the thermal stability of JAK1 after treatment with BLI. (G) Results of DARTS showing the protease stability of JAK1 after treatment with BLI. (H) A cell viability assay was used to analyze the biological activity of bio‐BLI (*n* = 3). (I) Pull‐down assay showing the binding of Bio‐BLI to JAK1 (*n* = 2). (J) The IC_50_ of BLI inhibiting JAK1 at 3.92 µM ATP concentration (*n* = 3). Significant *p* values are indicated on figure panels.

To further explore the molecular mechanisms of action of BLI, we conducted a transcription factor enrichment analysis using transcriptomic data. As shown in Figure 6A, the transcription factor STAT was the second most significantly altered factor. We also found that BLI significantly suppressed FA‐ or UUO‐induced phosphorylation of STAT1 and STAT3 (Figure 6B and Figure S7A). Furthermore, gene set enrichment analysis of transcriptomic data revealed that BLI significantly downregulated the IL6‐JAK‐STAT signaling pathway (Figure S7B). Therefore, we speculated that BLI might exert its effects by binding to STAT proteins or their upstream proteins. To explore the interaction between BLI and proteins, molecular dynamics stimulation was used. Figure 6C shows that the lowest binding energies occurred between BLI and STAT1, STAT3, JAK1, JAK2, and JAK3. We found that when BLI had the lowest binding energy with JAK1, it could bind to JAK1 via hydrogen bond interactions with Gly1020, Arg1007, and Gly884 (Figure 6D). Moreover, BLI inhibited JAK1 phosphorylation in mouse kidney tissues (Figure 6E and Figure S7C). Therefore, we hypothesized that BLI exerts a nephroprotective effect by binding to JAK1 and inhibiting its phosphorylation.

Cellular thermal shift assay (CETSA) and drug affinity responsive target stability (DARTS) are biophysical technologies used to assess the binding of a drug to its target protein.^38^, ^39^ The binding of a ligand to a target protein leads to an increase in thermal and protease stabilization. The DARTS results also indicated that BLI stabilized JAK1 in cell lysates after treatment with pronase (Figure 6F). The results of CETSA demonstrated that, compared with DMSO, BLI substantially enhanced the thermal stabilization of JAK1 at 54°C and 57°C in HK2 and NRK‐52E cells (Figure 6G). Next, we synthesized biotinylated BLI (Bio‐BLI, Scheme S1) and confirmed that its activity was similar to that of BLI (Figure 6H). A subsequent pull‐down assay using Bio‐BLI further confirmed the binding of BLI to JAK1 (Figure 6I). Finally, the effect of BLI on JAK1 kinase was evaluated,^40^ the results indicated that BLI is a potent inhibitor of JAK1 with an IC_50_ of 0.376 ± 0.0311 µM. Taken together, these results demonstrated that BLI can directly target and inhibit JAK1.

### 2.6 BLI inhibits ferroptosis by targeting JAK1 and inhibiting the JAK‐STAT signaling pathway

Numerous reports have provided compelling evidence for the regulatory role of the JAK‐STAT pathway in ferroptosis.^41^ Therefore, we hypothesized that BLI inhibits ferroptosis by modulating the JAK‐STAT signaling pathway. To further test this hypothesis, we used the JAK1 inhibitor Jak1‐In8 and the JAK1 agonist RO8191 in subsequent research. With respect to the Era‐induced ferroptosis model, we found that the JAK1 inhibitor Jak1‐In8 inhibited ferroptosis in a manner similar to that of BLI. However, when we combined the JAK1 agonist RO8191 with BLI, BLI was not effective (Figure S8A). Other indicators related to ferroptosis, such as iron ion levels, ROS, GSH, and MDA, were also measured. BLI and Jak1‐In8 significantly downregulated iron overload induced by Era, reducing cellular ROS and MDA levels while upregulating GSH levels (Figures S8B–D and S9A–C). Moreover, as shown in Figure S8E, BLI effectively suppressed the phosphorylation of JAK1, STAT1, and STAT3, and these effects were abolished by RO8191. We further examined hepcidin (HAMP), a major regulator of systemic iron metabolism,^42^ ferroportin1 (FPN), the only recognized iron‐exporting transporter in mammals,^43^, ^44^ and SLC7A11, a ferroptosis suppressor.^45^, ^46^ The results showed that Era treatment could increase the expression of HAMP and decrease the expression of FPN and SLC7A11 at the mRNA and protein levels, while BLI or Jak1‐In8 treatment could block this process, and these effects could be completely reversed by RO8191. These results suggest that the inhibition of ferroptosis by BLI is JAK1 dependent (Figures S8E and S9D).

BLI was reported to be a cyclin‐dependent kinase (CDK) inhibitor or peroxisome proliferator‐activated receptor gamma (PPAR‐γ) agonist.^47^, ^48^ Therefore, we used a CDK inhibitor (20‐223) and a PPAR‐γ agonist (pioglitazone) to mimic the possible mechanism of BLI. We found that 20–223 and pioglitazone were unable to block Era‐induced ferroptosis in HK2 and NRK‐52E cells (Figure S8F), indicating that CDK inhibition and PPAR‐γ activation could not inhibit ferroptosis. Therefore, BLI is unlikely to function through these two targets, further suggesting that BLI may exert its nephroprotective effect by targeting JAK1 rather than through CDK or PPAR‐γ. Taken together, these results demonstrated that BLI inhibits ferroptosis by targeting the JAK‐STAT signaling pathway.

### 2.7 JAK1 knockdown attenuated the nephroprotective effect of BLI

**FIGURE 7:**
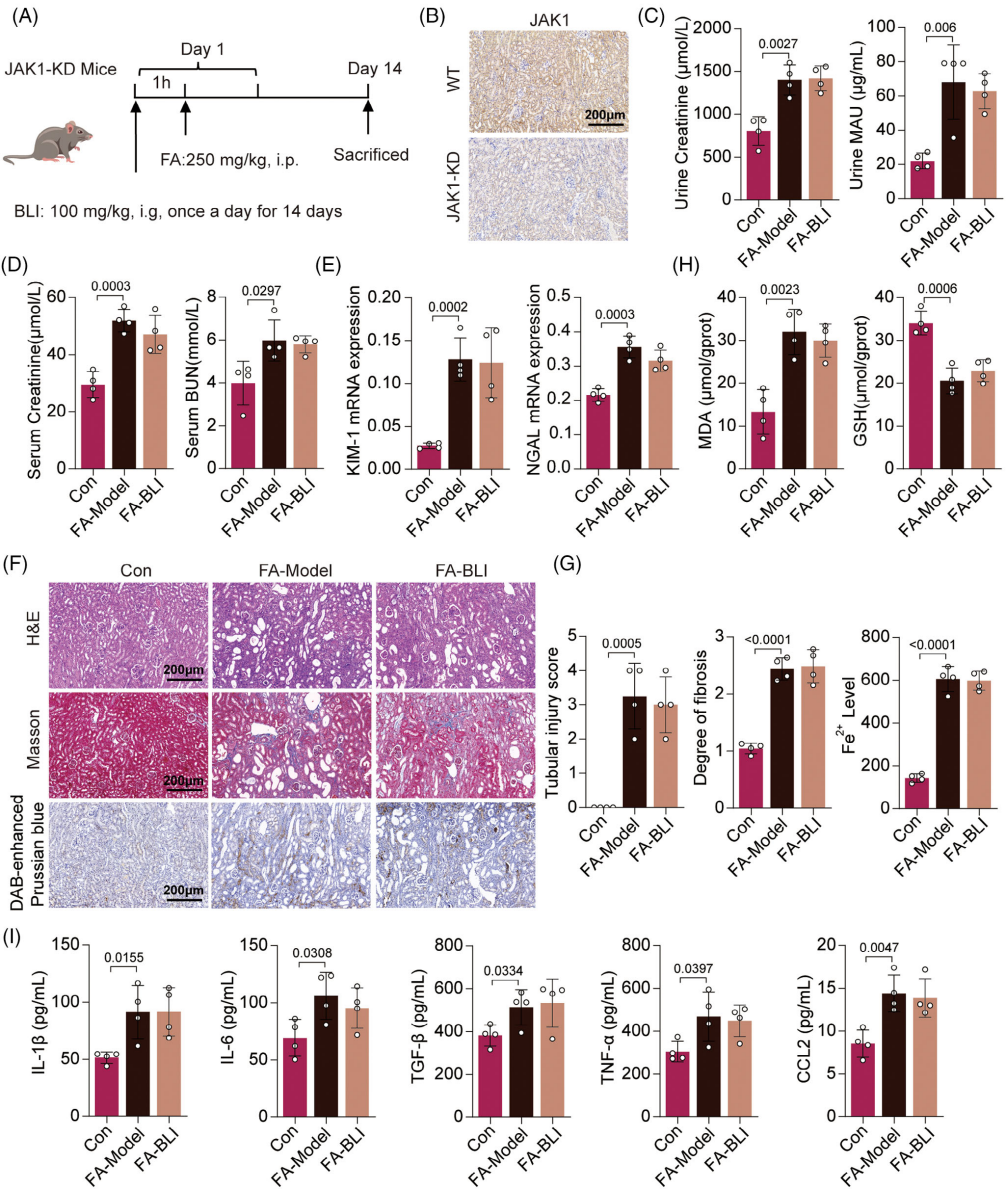
JAK1 knockdown attenuated the nephroprotective effect of BLI. (A) Scheme of the experiment. (B) Representative images of JAK1 staining of the kidneys (*n* = 4). (C) Urine levels of Cr and MAU (*n* = 4). (D) Serum levels of Cr and BUN (*n* = 4). (E) RT‐qPCR results of several kidney injury biomarkers (*n* = 4). (F) and (G) Representative images of H&E, Masson and DAB‐enhanced Prussian blue staining of the kidneys (*n* = 4). (H) Levels of MDA andGSH in kidneys (*n* = 4). (I) Serum levels of multiple chemokines (*n* = 4). Significant *p* values are indicated on figure panels. Scale bars were as shown in the figure.

To verify the role of JAK1 in the nephroprotective effect of BLI, we generated a gene‐edited mouse model in which JAK1 was knocked down (JAK1‐KD). JAK1‐KD mice were confirmed by immunohistochemistry and treated with FA and BLI as described (Figure 7A,B). We found that FA challenge increased kidney indices, serum Cr and BUN levels, and urinary Cr and MAU levels in JAK1‐KD mice. However, BLI treatment did not reduce the levels of these indicators (Figure 7C,D). Moreover, BLI treatment did not influence the mRNA expression of *KIM‐1* or *NGAL* (Figure 7E). Histological analysis also indicated that JAK1 knockdown attenuated the beneficial effects of BLI on pathological kidney injury, fibrosis, and iron concentration (Figure 7F,G). Finally, the serum levels of inflammatory cytokines and kidney tissue oxidative stress were also measured, and the results indicated that the anti‐inflammatory and antioxidant effects of BLI were diminished by JAK1 knockdown (Figure 7H,I). These results strongly suggest that BLI exerts its function dependent on JAK1 and exerts its nephroprotective effects by targeting JAK1.

### 2.8 BLI is as effective and safe as ivarmacitinib in AKI–CKD transition mouse model

**FIGURE 8:**
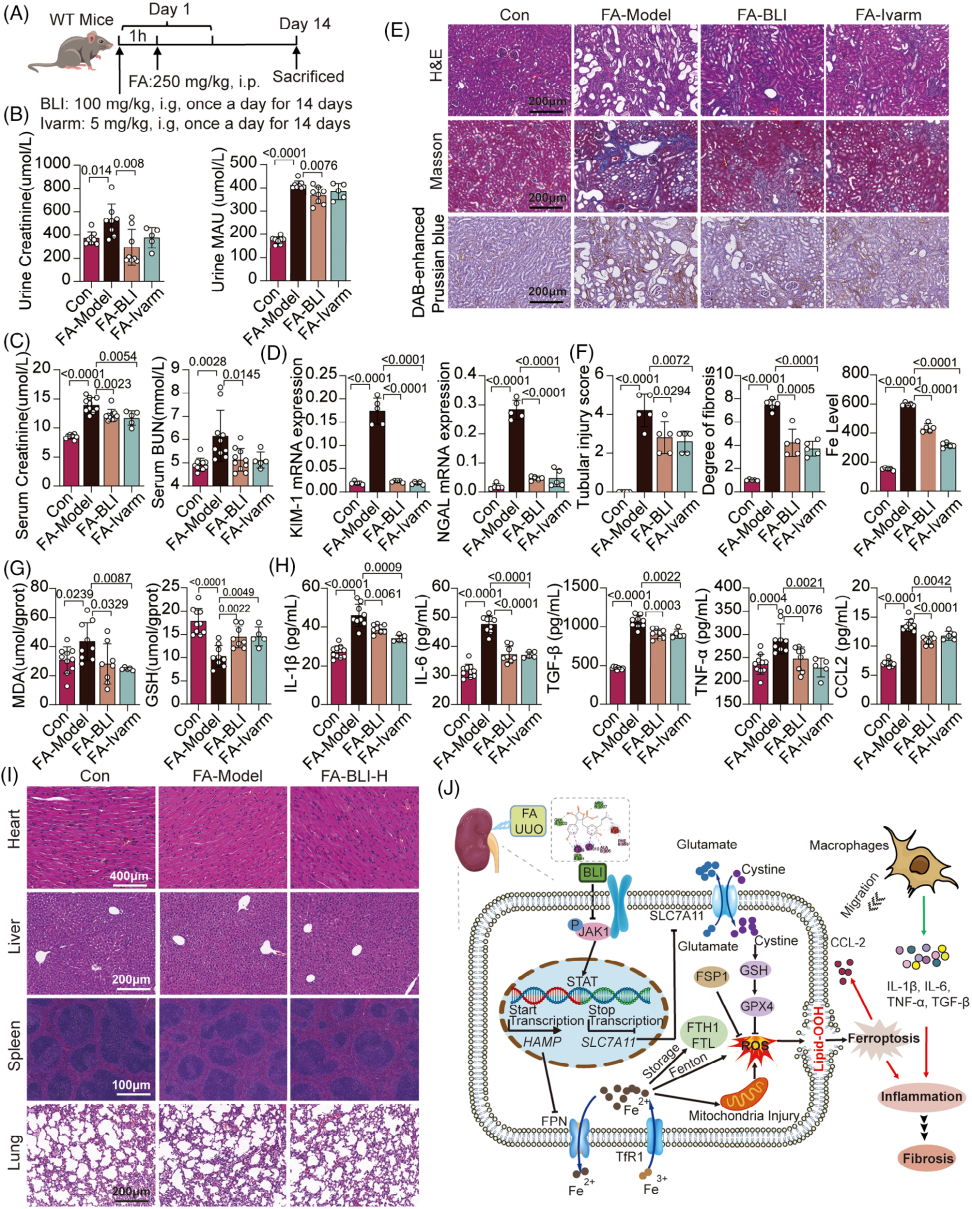
BLI is as effective and safe as ivarmacitinib in the AKI–CKD transition mouse model. (A) Scheme of the experiment. (B) Urine levels of Cr and MAU (*n* ≥ 5). (C) Serum levels of Cr and BUN (*n* ≥ 5). (D) RT‐qPCR results of several kidney injury biomarkers (*n* = 5). (E) and (F) Representative images of H&E, Masson, and DAB‐enhanced Prussian blue staining of the kidney (*n* = 5). (G) MDA and GSH levels in kidney tissues (*n* ≥ 5). (H) Serum levels of multiple chemokines (*n* ≥ 5). (I) H&E staining of mouse heart, liver, spleen, and lung tissues (*n* = 3). (J) Detailed molecular mechanism by which BLI blocks the AKI–CKD transition by targeting JAK1. Significant *p* values are indicated on figure panels. Scale bars were as shown in the figure.

Previous studies have confirmed that the JAK‐STAT signaling pathway is activated in CKD, especially in diabetic kidney disease (DKD), but JAK2 and STAT3 are the main concerns, and there are few studies on JAK1.^49^, ^50^, ^51^ To further demonstrate the possibility of alleviating CKD progression via targeted inhibition of JAK1, we used another specific JAK1 inhibitor, ivarmacitinib (Ivarm),^52^ in parallel with BLI (Figure 8A). We found that ivarmacitinib also effectively reduced kidney dysfunction, as well as the expression levels of ferroptosis biomarkers and serum inflammatory cytokines, similar to what was observed with BLI in the FA‐induced AKI–CKD transition model (Figure 8B–H). These results suggest that blocking AKI–CKD transition by targeting JAK1 is feasible and provide indirect evidence for the mechanism of nephroprotective effect of BLI.

We also preliminarily evaluated the toxicity of BLI in the FA‐induced AKI–CKD transition mouse model. The results of HE staining showed that mice in the untreated group and the high‐dose (100 mg/kg) BLI treatment group both exhibited clear tissue structures and intact morphology in the heart, liver, spleen, and lung, with no apparent damage. These results showed that BLI is safe and has almost no toxicity while playing a nephroprotective role in vivo (Figure 8I).

Taken together, our data strongly demonstrated the mechanism by which BLI blocks the AKI–CKD transition. For detail, BLI targets JAK1 and inhibits its phosphorylation and activation of the JAK‐STAT, subsequently regulating multiple downstream events: (1) promotes the transcription and translation of SLC7A11 to regulate the levels of intracellular GSH and GPX4, thereby maintaining the balance of the glutamate/cystine transport system and reducing ROS production; (2) inhibits the transcription and translation of HAMP, maintain iron homeostasis, and further inhibits iron deposition, mitochondrial damage, ROS production, lipid peroxidation, and ferroptosis; (3) inhibits the release of CCL2 and other factors, and reduces the number of macrophages recruited to the kidney tissue, thus inhibiting the inflammatory response characterized by increased levels of IL‐1β, IL‐6, TNF‐α, TGF‐β, and other factors. Finally, the AKI–CKD transition process is blocked, and the occurrence of kidney fibrosis is prevented (Figure 8J).

## 3 DISCUSSION

For decades, the cornerstone of pharmacologic therapy for CKD in the clinic has been RAS) blockade,^3^ and other therapeutic methods have focused mainly on the control of related pathogenic factors. However, even when underlying conditions are controlled, kidney damage can continue to worsen until complete or near‐complete kidney failure occurs and requires frequent dialysis or kidney transplants. There are no therapeutic interventions available to completely limit damage, accelerate recovery, or improve the survival rate.^1^ As a result, the treatment of CKD is very challenging and is currently considered to be incurable,^1^, ^6^, ^7^ and there is an urgent need to strengthen the development of related new drugs. An increasing number of studies have confirmed the role of AKI in the occurrence and progression of CKD.^53^ Timely intervention after AKI, that is, blocking the AKI–CKD transition, is an important consensus in the field of CKD treatment. Herein, a fresh attempt was made for the early discovery of drugs to block the AKI–CKD transition. We present a novel discovery: in mouse models of AKI–CKD transition, BLI demonstrates a nephroprotective effect that is comparable to or even superior to telmisartan in multiple indicators. Additionally, we disclose its mechanism of action. Notably, this is the first time we have demonstrated that BLI exerts pharmacological effects by targeting JAK1.

Clinically, kidney disease can be roughly divided into two categories, AKI and CKD, which are closely related. All the evidence from clinical and animal models supports the idea that AKI is closely related to the occurrence of CKD. Persistent AKI can induce interstitial fibrosis, failed cell differentiation, and ultimately CKD. It should be noted that there is a transitional phase between AKI and CKD, namely, the AKI–CKD transition, which has been explored in several studies. The reliability and clinical applicability of our findings are mainly supported by two classic mouse models of the AKI–CKD transition. We first used the FA‐induced AKI–CKD transition model to evaluate the nephroprotective effect of BLI because this model is reproducible and can recapitulate most, if not all, of the human AKI and CKD phenotypes.^54^ We also used the UUO‐induced AKI–CKD transition model, which is widely used to study obstructive nephropathy that can lead to AKI and CKD.^21^ For the two mouse models used in this study, it is generally believed that the AKI stage occurs 3–7 days after modeling, the AKI–CKD transition stage immediately occurs, and the CKD stage occurs 14 days after modeling and above.^54^, ^55^, ^56^ We stopped the experiment and tested kidney indicators after 14 days, and the results showed that BLI improved the kidney function of mice that had entered the CKD stage after the AKI–CKD transition, which fully indicated that the neuroprotective effect of BLI included the inhibition of AKI and the subsequent AKI–CKD transition process. Specifically, BLI treatment can inhibit the AKI‐induced inflammatory response and kidney fibrosis. Extensive evidence suggests that kidney inflammation plays a central role in the occurrence and development of CKD and that kidney fibrosis is the ultimate common pathway of progressive kidney disease.^1^, ^25^, ^55^, ^57^, ^58^ These data strongly support the nephroprotective effect on the AKI–CKD transition process and the clinical application potential of BLI.

To explore how BLI works, we performed RNA sequencing analysis and other methods and ultimately verified that ferroptosis inhibition plays an important role in the nephroprotective effect of BLI. Ferroptosis has received increasing attention because emerging evidence indicates that ferroptosis is correlated with the occurrence and development of various diseases, including AKI and CKD.^29^, ^30^ We assessed the levels of several well‐established biomarkers of ferroptosis, such as iron, GSH, and MDA, as well as GPX4, FSP1, TfR1, FTH1, FTL, and SLC7A11. These results strongly prove that BLI can inhibit ferroptosis induced by AKI. Furthermore, using a small molecule (Era) that inhibits SLC7A11, we established a kidney cell model of ferroptosis. Consistent with the mouse results, BLI significantly inhibited Era‐induced ferroptosis in the cell model.

After confirming that BLI has good ferroptosis inhibition activity, we searched for potential targets of BLI. Previous studies have reported that BLI is an inhibitor of CDK2 and CDK5, as well as a partial agonist of PPAR‐γ.^47^, ^48^ However, in our study, we found that BLI does not exert its inhibitory effect on ferroptosis through these targets. Subsequently, we performed transcription factor enrichment analyses using transcriptomic data and further validated the results using CETSA, DARTS, and pull‐down experiments, the results of which demonstrated that there is a direct interaction between BLI and JAK1. In addition, we used JAK‐KD mice to construct a CKD model and demonstrated that the nephroprotective effect of BLI is JAK1 dependent, inversely indicating that JAK1 is the target of BLI. Previous studies have shown that enhanced JAK‐STAT expression and activation occur in many kidney diseases and different CKD models, the most studied of which is DKD.^49^, ^50^, ^51^ Clinical trials using JAK inhibitors to treat DKD have also shown promising results.^59^ Our results are consistent with these existing findings and further demonstrate that CKD can be treated by targeting JAK and its associated signaling pathways. The most common activation subtypes in CKD are JAK2 and STAT3, which play a role in damaging processes such as persistent inflammation and fibrosis.^49^ Our findings focused on JAK1 and demonstrated the role and mechanism of JAK1 as a therapeutic target using two AKI–CKD transition models, expanding the scope of research in this field and providing a reference for subsequent relevant studies.

Many studies have shown that activation of the JAK‐STAT signaling pathway leads to SLC7A11 transcriptional inhibition and enhanced ferroptosis.^44^, ^45^ Consistent with these findings, our study revealed that BLI could downregulate SLC7A11 by inhibiting the JAK1‐STAT signaling pathway. Moreover, when the JAK1 agonist RO8191 was used, the regulatory effect of BLI on SLC7A11 was suppressed. The HAMP‐FPN axis is the principal regulator of extracellular iron homeostasis in health and disease and is a promising target for the diagnosis and treatment of iron disorders. The expression of HAMP is regulated by two main signaling pathways: the iron‐regulated bone morphogenetic protein pathway and the inflammatory JAK‐STAT pathway.^60^ The JAK/STAT signaling pathway induces the expression of HAMP by acting through a conserved STAT‐binding motif located near the transcription start site. With respect to the Era‐induced ferroptosis model, we discovered that the JAK‐STAT signaling pathway was significantly activated. The expression of HAMP significantly increased, while that of FPN decreased. This leads to an increase in cellular iron ion levels. However, treatment with BLI suppressed the JAK‐STAT pathway, leading to decreased HAMP expression and increased FPN protein levels. Taken together, these results not only demonstrated that JAK1 is the target of BLI but also provided a reference for basic research on JAK1 and the JAK‐STAT signaling pathway.

This study has several limitations, which we should consider in future research. For example, the mechanism by which BLI targets JAK1 for nephroprotection may be far more complex than we have discovered, and this mechanism needs to be studied in depth with more advanced techniques and models in the future. In addition, the effects of BLI on animal models of progressive CKD and its metabolism are important for evaluating its potential as a drug candidate and are among the focuses of our future research. The use of BLI in other CKD types, such as DKD, is also worthy of further study.

In conclusion, our study demonstrated that BLI has significant nephroprotective effects, including maintenance of kidney function, inhibition of the inflammatory response, and prevention of fibrosis. Mechanistically, BLI works primarily by targeting JAK1 to inhibit the JAK‐STAT signaling pathway and subsequent ferroptosis in AKI–CKD transition models. Our findings suggest that BLI may be a potential candidate for the treatment of CKD, which is worthy of further investigation and could hopefully lead to new possibilities for CKD drug development.

## 4 MATERIALS AND METHODS

### 4.1 Animal experiments

Male C57BL/6J mice (8 weeks, 22–24 g) were purchased from Vital River (Beijing, China, Certificate No.: SCXK2022‐0030). JAK1‐KO C57BL/6 mice were purchased from Gempharmatech Co., Ltd. (Shanghai, China, Certificate No.: SCXK 2023‐0009). The mice were housed under specific pathogen‐free conditions and were provided free access to tap water and rodent food and were fed under controlled conditions. After 3 days of adaptation to the housing conditions, the mice (C57BL/6J) were randomly divided into five groups as follows (*n* ≥ 10/group): Con (0.3 mM NaHCO_3_), model (250 mg/kg FA), BLI‐L (250 mg/kg FA + 50 mg/kg BLI), BLI‐H (250 mg/kg FA + 100 mg/kg BLI), and Tel (5 mg/kg Tel) groups. The mice were sacrificed 14 days after FA treatment, and kidney, blood, and urine specimens were collected for further experiments.

### 4.2 Detection of kidney function parameters

Whole‐blood samples were allowed to clot for 1 h at 4°C before centrifugation at 3500 × *g* for 15 min for collection of serum. Cr, BUN, urine Cr, and MAU were measured using a specific reagent kit from Nanjing Jiancheng Bioengineering Institute (Nanjing, China).

### 4.3 RNA‐seq profiling

Total RNA was extracted from kidney tissues with TRIzol reagent (Biosharp). Three biological replicates for the Con group, Model group, and BLI group were sequenced using the BGISEQ‐500 platform. The DEGs were screened out if they had a *q* value ≤ 0.001 and a fold change ≥2. Kyoto Encyclopedia of Genes and Genomes (KEGG) pathway functional enrichment analysis was subsequently performed to reveal the pathways influenced by BLI. Transcription factor analysis was performed to identify the transcription factors influenced by BLI.

### 4.4 Cell lines and culture

HK2 and NRK‐52E cells were obtained from Procell Life Science & Technology Co., Ltd. HK2 cells were maintained in minimum essential medium (HyClone) supplemented with 10% fetal bovine serum (FBS), 1% nonessential amino acids, 100 U/mL streptomycin, and 100 U/mL penicillin (Gibco). NRK‐52E cells were maintained in Dulbecco's modified Eagle's medium (containing 4.5 g/L d‐glucose) supplemented with 5% FBS, 100 U/mL streptomycin, and 100 U/mL penicillin. Both cell lines were cultured in a 37°C incubator with 5% CO_2_.

### 4.5 JAK1‐BLI binding assays

The binding of BLI to JAK1 was determined by multiple assays. HK2 and NRK‐52E cells were seeded in 10‐cm culture dishes until they reached 80%–90% confluence. For the CETSA, prior to cell collection, the cells were treated with BLI or the same volume of vehicle for 90 min. Then, the cells were collected and lysed in RIPA lysis buffer for 20 min, and the supernatant was collected by centrifugation at 10,000 × *g* at 4°C for 10 min. The lysates were divided into PCR tubes and heated at a designated temperature (42–57°C) for 3 min on a heating block. After heating, the tubes were placed on ice for 3 min and boiled in loading buffer at 95°C for 10 min for immunoblotting analysis.

For the DARTS assay, the cells were collected and lysed in RIPA lysis buffer for 20 min. After that, the supernatant was collected by centrifugation at 10,000 × *g* at 4°C for 10 min. The lysates were divided into PCR tubes in equivalent volumes containing 100 µg of protein and incubated with BLI or the same volume of vehicle for 20 min at 4°C. Then, pronase (Sigma) was added to the samples. The samples were incubated at 25 °C for 10 min. Finally, the lysates were boiled in loading buffer at 95°C for 10 min for immunoblotting analysis.

For the pull‐down assay, biotinylated BLI was added to streptavidin‐agarose beads (Thermo Fisher Scientific) and incubated at 4°C for 8 h. Biotin alone, unbiotinylated BLI, and untreated beads were used as controls. Lysates prepared from HK2 and NRK‐52E cells were then added to streptavidin‐agarose beads with Bio‐BLI. The mixture was incubated at 4°C for 1 h with gentle rocking. The samples were washed three times and boiled in loading buffer at 95°C for 10 min for immunoblotting analysis.

### 4.6 JAK1 kinase activity assay

JAK1 kinase was obtained from Invitrogen, and assay was performed using HTRF (Homogenous Time Resolved Fluorescence) detection technology.^40^ Briefly, the enzyme reaction was conducted in reaction buffer consisting of 1 mM DTT, 5 mM MgCl_2_, 50 mM HEPES (pH7.0), 0.05% BSA, and 0.1% NaN_3_. The assay was performed in a 384‐well plate (10 µL) assay format. The final concentrations of the enzyme, TK‐substrate‐biotin, and ATP were 1 ng/µL, 1 µM, and 3.92 µM, respectively. Compounds were screened at serial diluted concentrations in the presence of 2% DMSO with a 5 min pre‐incubation of the kinase and compounds. All reactions were initiated by the addition of ATP and TK‐substrate‐biotin, incubated at 30°C for 30 min, and quenched with the stop buffer containing 25 nM Strep‐XL665 and TK Ab‐Cryptate. Time‐resolved fluorescence was monitored using a Synergy H1 (Biotek) by excitation at 330 nm and emission donor at 620 nm or emission acceptor at 665 nm, respectively. The files recorded by the Synergy H1 were analyzed in Excel and contained the acceptor and donor counts for each sample. IC_50_ values were determined using the Graphpad Prism 10 Software.

### 4.7 Statistical analysis

The data are expressed as the mean ± SEM. For comparisons between two groups, statistical significance was determined using Student's *t* test. When comparing more than two groups, one‐way analysis of variance was used to assess statistical significance. A *p* value less than 0.05 was considered to indicate statistical significance. Other methods and materials related to the fermentation, isolation, identification of BLI, and synthesis of biotinylated BLI are included in the Supporting Information.

## AUTHOR CONTRIBUTIONS

Yonghui Zhang and Qingyi Tong conceived the project. Zijun Zhang and Ziming Zhao performed most of the experiments. Changxing Qi and Xiaotian Zhang isolated and extracted the BLI. Tiantai Zhang and Chengjuan Chen were responsible for the activity detection experiment of JAK1. Qingyi Tong and Ziming Zhao analyzed the data and wrote the manuscript. Zijun Zhang prepared the figures. Yu Zou was responsible for the biotin modification of the BLI. Yang Xiao, Xia Chen, Lianghu Gu, and Jianzheng Huang participated in some of the experiments. Kun Huang and Ming Xiang participated in the project discussion and gave many useful suggestions. All authors have read and approved the final manuscript.

## CONFLICT OF INTEREST STATEMENT

The authors declare no conflicts of interest.

## ETHICS STATEMENT

The Animal Experiment Administration Committee of the Huazhong University of Science and Technology (HUST) approved all animal experiments to ensure ethical and humane treatment of animals (2023 IACUC Number: 3612).

## Supporting information



Supporting Information

## Data Availability

The raw sequence data reported in this paper have been deposited in the Genome Sequence Archive (Genomics, Proteomics & Bioinformatics 2021) in National Genomics Data Center (Nucleic Acids Res 2022), China, and National Center for Bioinformation/Beijing Institute of Genomics, Chinese Academy of Sciences (GSA: CRA016360) that are publicly accessible at https://ngdc.cncb.ac.cn/gsa.
