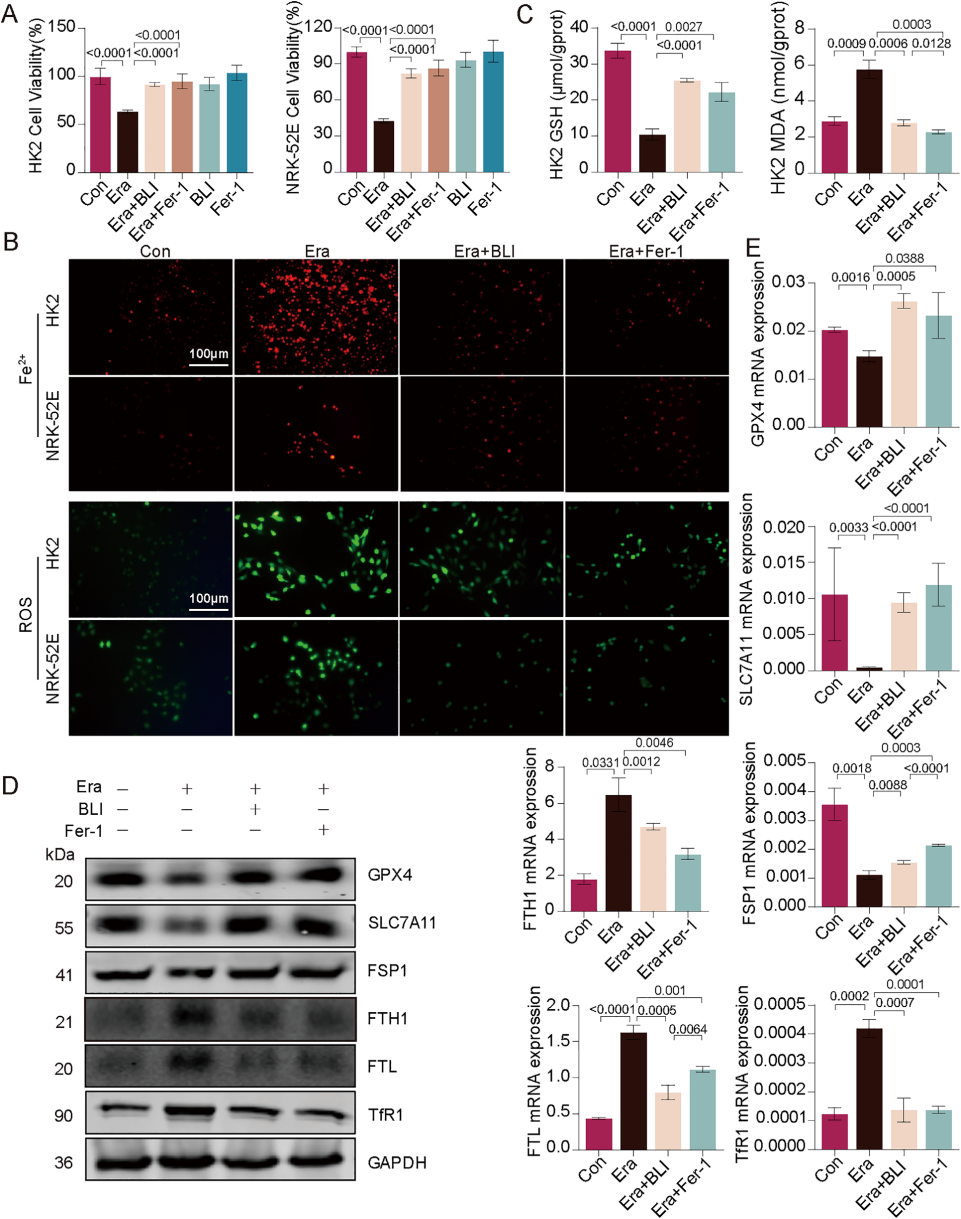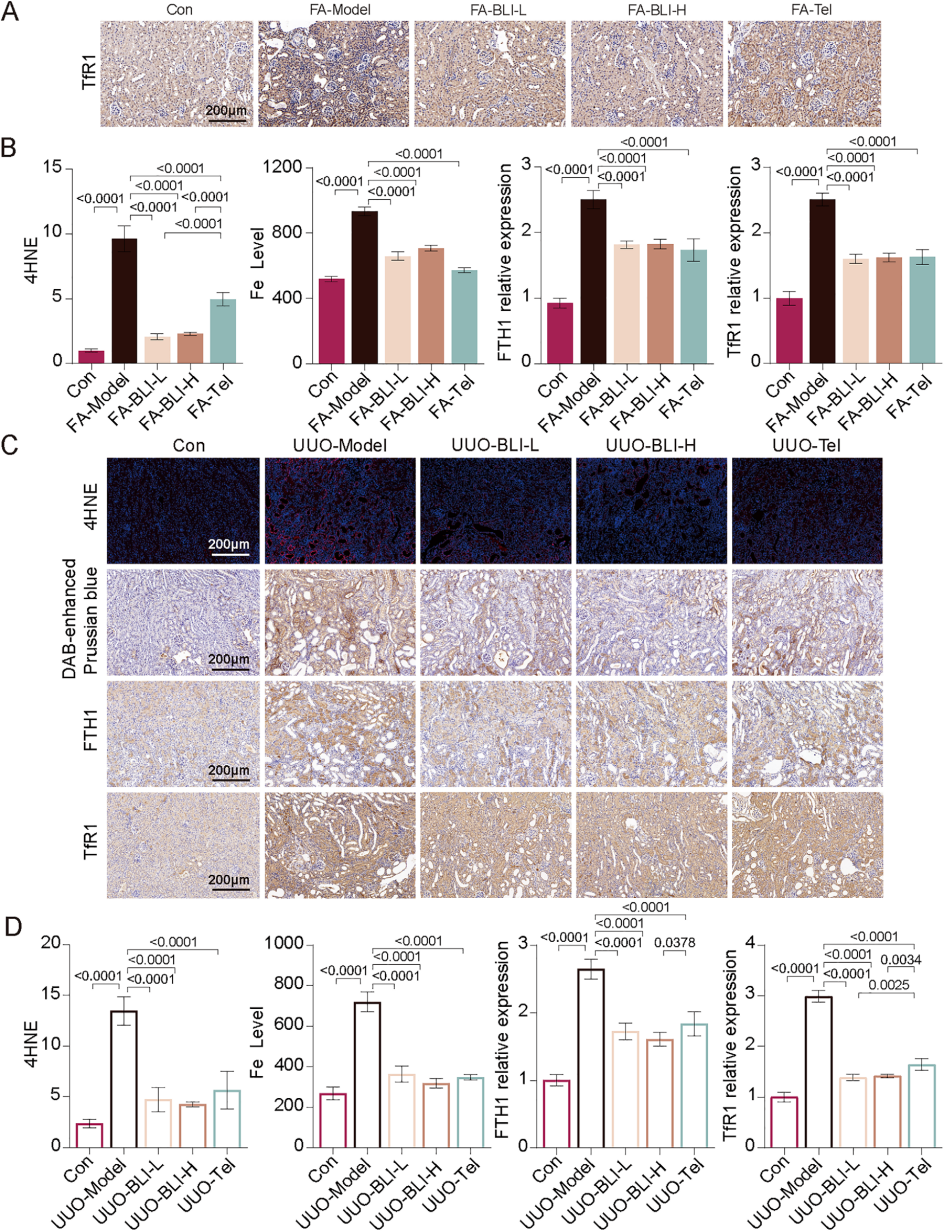# Correction to “Butyrolactone I Blocks the Transition of Acute Kidney Injury to Chronic Kidney Disease in Mice by Targeting JAK1”

**DOI:** 10.1002/mco2.70552

**Published:** 2025-12-09

Zijun Zhang, Ziming Zhao, Changxing Qi, Xiaotian Zhang, Yang Xiao, Chengjuan Chen, Yu Zou, Xia Chen, Lianghu Gu, Jianzheng Huang, Kun Huang, Ming Xiang, Tiantai Zhang, Qingyi Tong, Yonghui Zhang. Butyrolactone I blocks the transition of acute kidney injury to chronic kidney disease in mice by targeting JAK1. MedComm, 2025, 6(2): e70064.

In Figure 5B (Results 2.4, page 9), due to the lack of prompt and accurate saving during the image processing procedure, the control image for ROS staining of NRK‐52E cells was inadvertently placed over that of the HK2 cell control group; this has now been corrected. Similarly, in Figure S5C (Supporting Information, page 9), the 4HNE staining image for the UUO‐BLI‐H group was superimposed over that of the UUO‐Tel group, which has also been rectified.

We apologize for these errors.