# Supplementary material for: Butyrolactone I blocks the transition of acute kidney injury to chronic kidney disease in mice by targeting JAK1
Source: MedComm (2020). 2025 Jan 21;6(2):e70064. doi: 10.1002/mco2.70064 (PMC11751251; doi:10.1002/mco2.70064)
Supplement: Supplementary file 1 — Supporting Information [file MCO2-6-e70064-s001.docx]

**Butyrolactone I blocks the transition of acute kidney injury to chronic kidney disease in mice by targeting JAK1**

Zijun Zhang^1#^, Ziming Zhao^1#^, Changxing Qi^1#^, Xiaotian Zhang^1#^, Yang Xiao^1^, Chengjuan Chen^2^, Yu Zou^3^, Xia Chen^3^, Lianghu Gu^1^, Jianzheng Huang^1^, Kun Huang^1^, Ming Xiang^1^, Tiantai Zhang^2^, Qingyi Tong^1^*, Yonghui Zhang^1^*

Author Affiliations: 1. Hubei Key Laboratory of Natural Medicinal Chemistry and Resource Evaluation, School of Pharmacy, Tongji Medical College, Huazhong University of Science and Technology, Wuhan, Hubei, China. 2. State Key Laboratory of Bioactive Substances and Function of Natural Medicine, Institute of Materia Medica, Chinese Academy of Medical Sciences, Peking Union Medical College, Beijing, China

3.Institute of Pharmaceutical Process, Hubei Province Key Laboratory of Occupational Hazard Identification and Control, School of Medicine, Wuhan University of Science and Technology, Wuhan 430065, China.

*Correspondence: Yonghui Zhang and Qingyi Tong; Tel.: +86-27-8369286; Fax: +86-27-83692762. E−mail: zhangyh@mails.tjmu.edu.cn and [qytong@hust.edu.cn](mailto:qytong@hust.edu.cn).

^#^These authors made equal contributions to this work.

**1. Supplementary** **materials and methods**

**Fungal material**

The fungus *A. terreus* was isolated from the soil at the bottom of the Yangzi River in May 2013. The sequence data for this strain have been submitted to the DDBJ/EMBL/GenBank under accession number KT360948. A voucher sample, QCX20130513, was preserved in the herbarium of Tongji Medical College, HUST.

**Fermentation and isolation of butyrolactone I (BLI)**

To prepare the seed culture, the strain was cultured on potato dextrose agar (PDA) at 28 ℃ for 7 days. The agar plugs were cut into small pieces (approximately 0.6 × 0.6 × 0.6 cm^3^). Then, the strain was inoculated into 50 Erlenmeyer flasks (5 L) that were previously sterilized by autoclaving, and each flask contained 1000 g of rice and 1000 mL of distilled water. The flasks were incubated at 28 ℃ for 28 days. After incubation, the growth of the fungus was stopped by adding 1000 mL of ethanol to each flask, followed by extraction with ethanol. We used reduced pressure to remove the ethanol and yielded a brown extract (880.0 g). The extracts were subjected to silica gel chromatography (CC) and eluted with CH_2_Cl_2_/MeOH (10:1–1:1) to obtain six fractions (Fr. 1–Fr. 6). We separated Fr. 3 with repeated silica gel CC to yield three subfractions (Fr. 3.1–Fr. 3.3) and then subjected subfraction Fr. 3.2 to Sephadex LH-20 CC (CHCl_3_–MeOH, 1:1) to afford four fractions (Fr. 3.2a–Fr. 3.2d). Fr. 3.2b was purified by recrystallization on dichloromethane to yield a high yield of BLI (556.2 g).

**Identification and purity detection of BLI**

According to the literature (Rao, K.V., Sadhukhan, A.K., Veerender, M., Mohan, E.V.S., Dhanvantri, S.D., Sitaramkumar, S., Babu, M.J., Vyas, K., Reddy, O.G., Butyrolactones from *Aspergillus terreus*. Chem. Pharm. Bull., 2000, 48, 559–562), we confirmed that our isolated compound was BLI (Figure S1). In addition, the purity of the isolate was evaluated on an Agilent 1200 system and a Dionex HPLC system with a reversed-phase (RP) C_18_ column (3 *µ*m, 4.6×250 mm, Welch Ultimate AQ-C_18_) at a flow rate of 1 mL/min and a MeOH−H_2_O ratio of 30:70 (v:v). The results showed that the purity of the used BLI in this study was >99%.

**Synthesis of biotinylated BLI**

**Scheme 1. Synthesis of biotinylated BLI.** BLI (0.5 g, 1.19 mmol, 1 eq), SM2 (0.435 g, 1.78 mmol, 1.5 eq), EDCI-HCI (0.34 g, 1.78 mmol, 1.5 eq) and HOBt (0.24 g, 1.78 mmol, 1.5 eq) were added to 3 mL of DMF at room temperature with nitrogen protection. The reaction mixture was stirred for 2 h at 25 °C until the raw material point basically disappeared. The reaction was stopped by adding 50 mL of DCM, and the mixture was washed five times with water (15 mL of water each time). After washing, anhydrous sodium sulfate was added to the DCM layer to remove the water, and the filtrate was drained and weighed. Isopropyl ether was added three times (2-3 mL of isopropyl ether was added each time), the isopropyl ether phase was discarded, DCM and 0.5 g of silica gel were added to make the sand, and 4-5 g of silica gel was added to the column. With a column chromatography ratio of PE/EA=1:3, the raw material was washed to no fluorescence and then eluted with DCM/MeOH=20:1 to obtain the product, which was dried under a solid vacuum.

**Detection of MDA and GSH levels**

The individual levels of GSH and MDA were measured using GSH and MDA kits purchased from Nanjing Jiancheng Bioengineering Institute (Nanjing, China) according to the manufacturer’s protocol.

**Histology analysis**

Kidney tissues were fixed with 4% paraformaldehyde for 12 hours and embedded in paraffin. The paraffin-embedded kidney specimens were sectioned (4 μm) and stained with H&E, Masson trichrome, DAB-enhanced Prussian blue and4-HNE according to the manufacturer’s protocol. Other sections of kidney specimens were subjected to immunohistochemistry (IHC), and the samples were incubated with primary antibodies against α-SMA (ProteinTech, Wuhan, China), Fn (ProteinTech), Col-I (Proteintech), CD68 (ProteinTech), F4/80 (ProteinTech), FTH1 (CST, USA), TfR1 (ProteinTech), and JAK1 (Abcam, UK) for 30 minutes at room temperature. Subsequently, the slides were incubated with secondary antibodies and hematoxylin.

**Cell viability assay**

HK2 and NRK-52E cells were plated in 96-well plates. The cells were incubated with Era (Sigma, USA) in the absence or presence of BLI and the other agents used for Fer-1 (Sigma), Jak1-In8 (MCE, New Jersey, USA), or RO8191 (RO; MCE) for 24 hours. One hundred microliters of 10% Cell Counting Kit-8 (CCK-8; Topscience, Shanghai, China) solution was added to each well. The plates were incubated at 37°C for 1 hour, after which the absorbance was measured at 450 nm by using a multimode microplate reader (Bio-Rad, Hercules, CA, USA).

**Intracellular iron determination**

After drug treatment for 24 hours, the cells were mixed with RhoNox-1 (5 μM; MCE, USA) and incubated in the dark at 37°C for 30 minutes. After the cells were washed with PBS twice, they were observed via fluorescence microscopy.

**Analysis of intracellular ROS levels**

After drug treatment for 24 hours, the cells were mixed with 2,7-dichlorofuorescin diacetate (2 μM; Nanjing Jiancheng Bioengineering Institute) and incubated in the dark at 37°C for 20 minutes. After the cells were washed with PBS twice, they were observed via fluorescence microscopy.

**Analysis of the intracellular mitochondrial membrane potential (MMP)**

The cells were plated on 12-well plates and treated the next day. For JC-1 (Nanjing Jiancheng Bioengineering Institute) imaging, the cells were washed twice with PBS 24 hours after treatment, stained with 2 μM JC-1 in incubation buffer for 15 minutes at 37°C and 5% CO_2_, and again washed twice in incubation buffer. The cells were imaged using a Nikon confocal microscope.

**Molecular docking**

The crystal structure of the protein was derived from the Protein Data Bank. The initial structure of the BLI (PubChem CID:123740) was accessed from the PubChem database. Molecular docking was performed with AutoDock software. The docking parameters were set as previously described (assessing molecular docking tools to guide targeted drug discovery of CD38 inhibitors).

**Immunoblotting**

Cells and kidney tissue were lysed with RIPA buffer containing 1× protease inhibitor cocktail (Beyotime, China) and 1 mM phenylmethylsulfonyl fluoride (PMSF; Beyotime) at 4°C for 20 minutes. Afterwards, the samples were centrifuged at 10000 × g and 4°C for 10 minutes, after which the supernatants were collected. The protein concentration was determined by a BCA kit (Beyotime). After that, the samples were loaded on a 10-15% SDS-PAGE gel and electrotransferred onto a nitrocellulose filter membrane (Pall Corporation, USA) with 20% methanol. The membranes were blocked with 5% milk in TBS buffer for 1 h at room temperature, followed by incubation with specific primary antibodies at 4°C overnight. The specific primary antibodies used in this study were against JAK1, p-JAK1, STAT1, p-STAT1, STAT3, p-STAT3, GPX4, SLC7A11, FSP, FTH1, FTL, TfR1, HAMP, α-SMA, COL-Ⅰ, COL-III, COL-IV, and Fn. Next, the membranes were washed three times with TBST buffer and incubated with the corresponding secondary antibody (DyLight™ 800 or DyLight™ 600-conjugated) at 37°C for 1 hour. Finally, the membranes were visualized by an Odyssey® CLx Imaging System (LICOR).

**Quantitative real-time PCR (qPCR)**

Total RNA was isolated from kidney tissues and HK2 cells with TRIzolTM Reagent (Thermo Fisher Scientific) according to the manufacturer’s directions, and cDNA was synthesized with HisScript® II Q RT SuperMix for q-PCR (Vazyme, Nanjing, China). Subsequently, the cDNA was amplified using SYBR Green Mix (Biosharp, Beijing, China). The normalization of relative gene expression was performed with respect to the levels of endogenous β-actin. The sequences of the primers used are listed in supplementary Table 1 and Table1 2.

**Safety comment**

No unexpected or unusually high safety hazards were encountered.

**2. Supplementary figures**


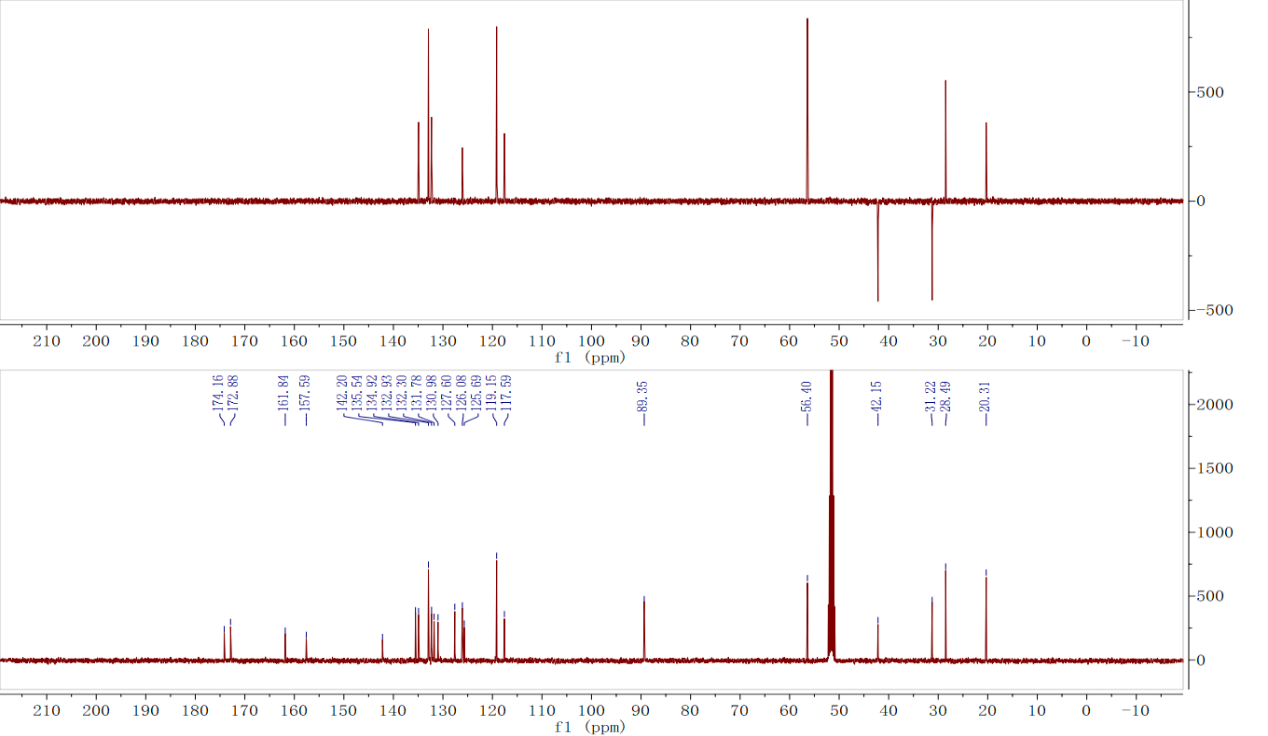
**Figure S1. ^13^C and DEPT NMR for BLI (400 MHz, 25 ℃, methanol-*d*_4_).**


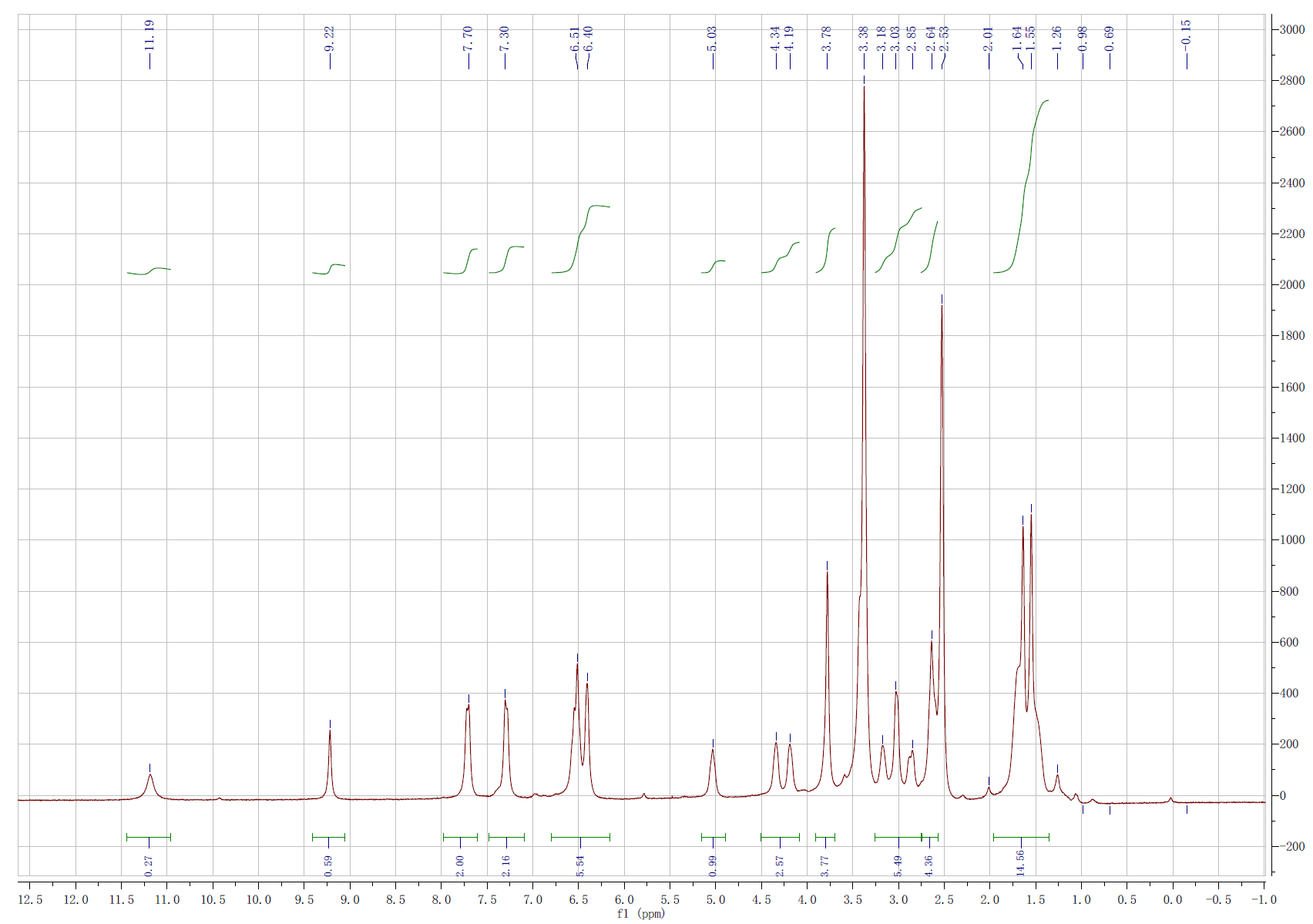


**Figure S2. ^1^H NMR for biotinylated BLI (400 MHz, 25 ℃, methanol-*d*_4_).**


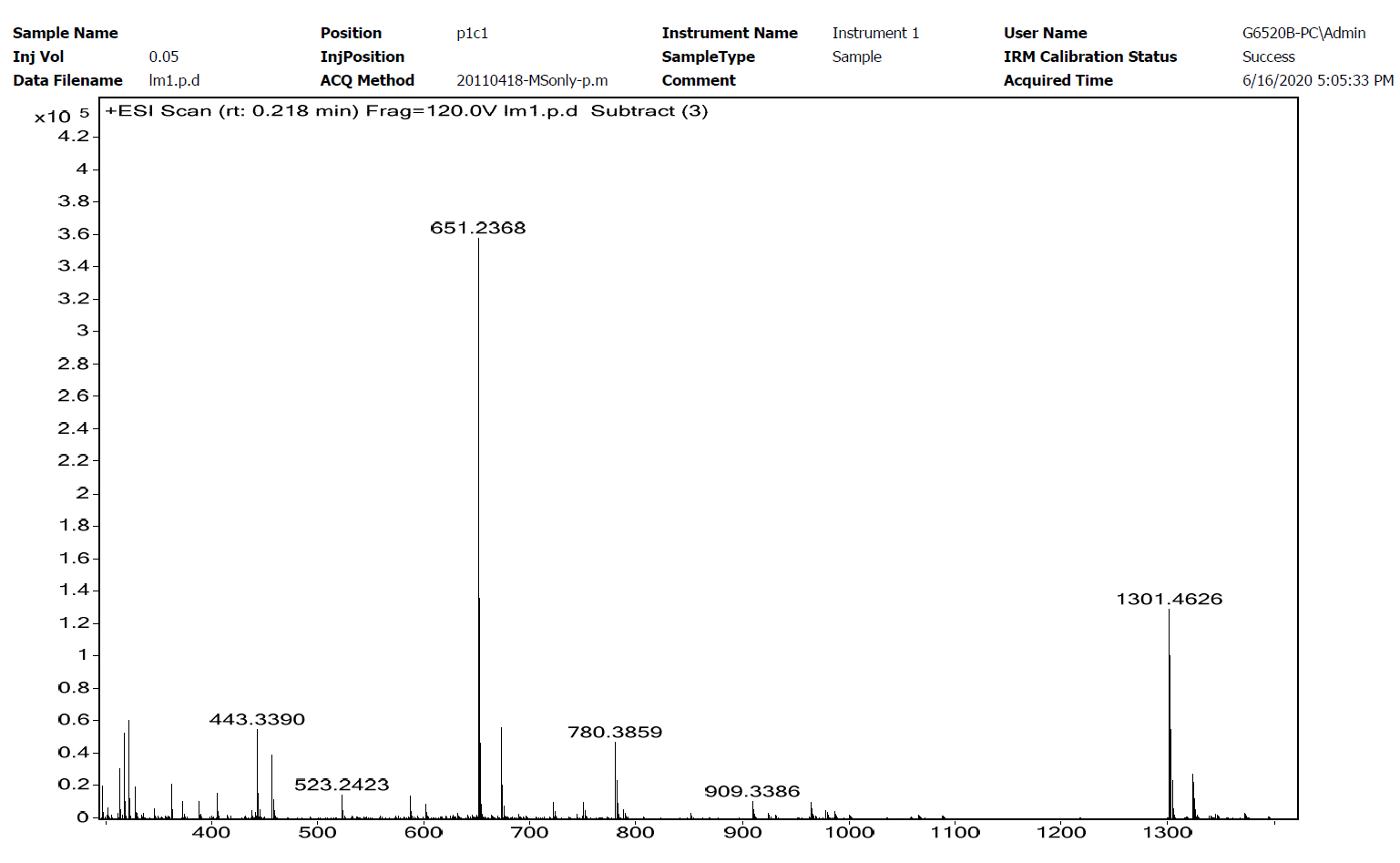


**Figure S3. HRESIMS spectrum for biotinylated BLI (*m*/*z* 651.2368 [M + H]^+^, calcd for 651.2376).**


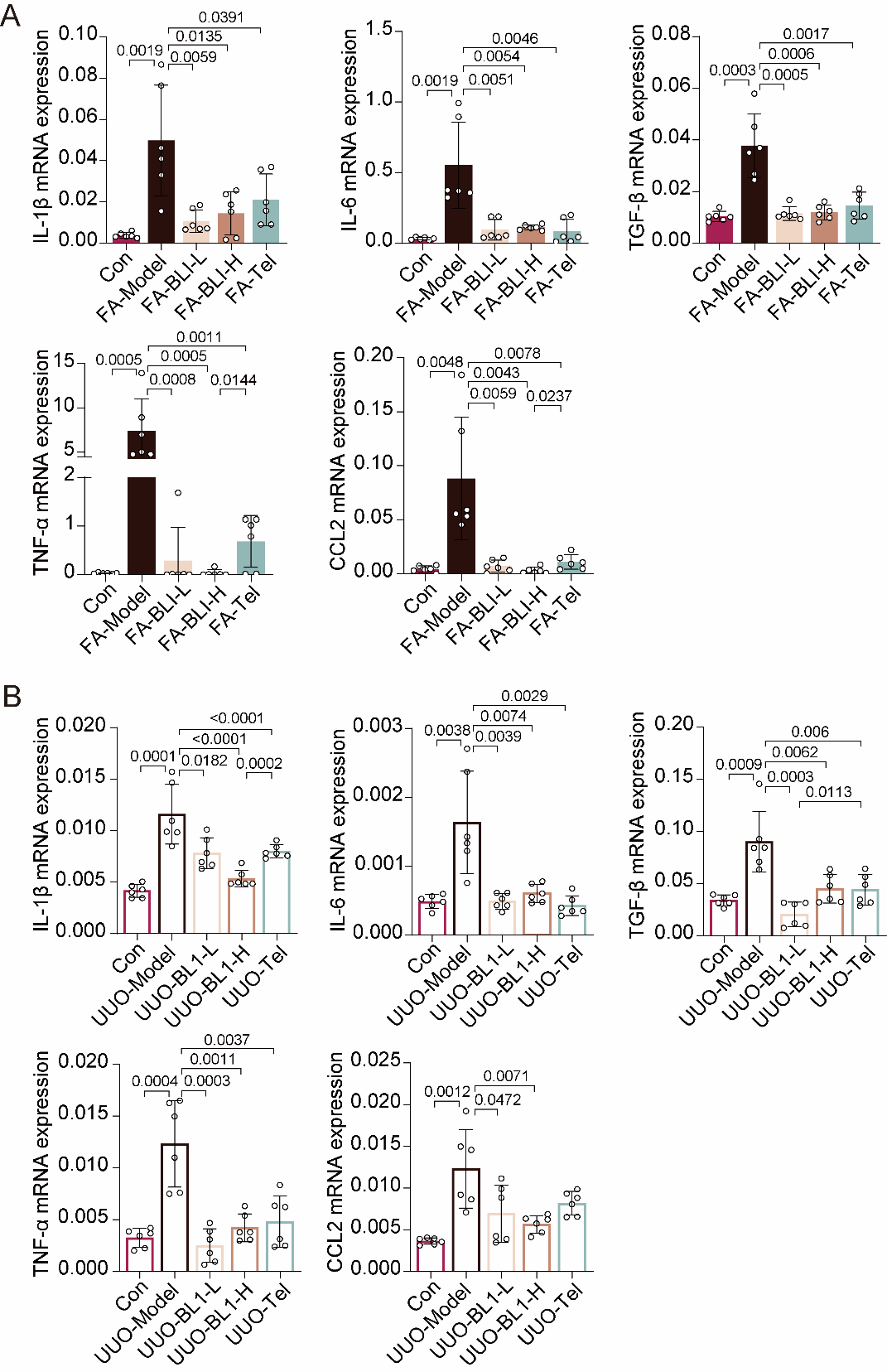


**Figure S4. BLI decreased the expression of inflammation-related chemokine genes induced by FA or UUO.** (A) and (B), mRNA levels of inflammation-related chemokine genes in the kidneys of the two mouse models (n=6). Significant P values are indicated on figure panels.


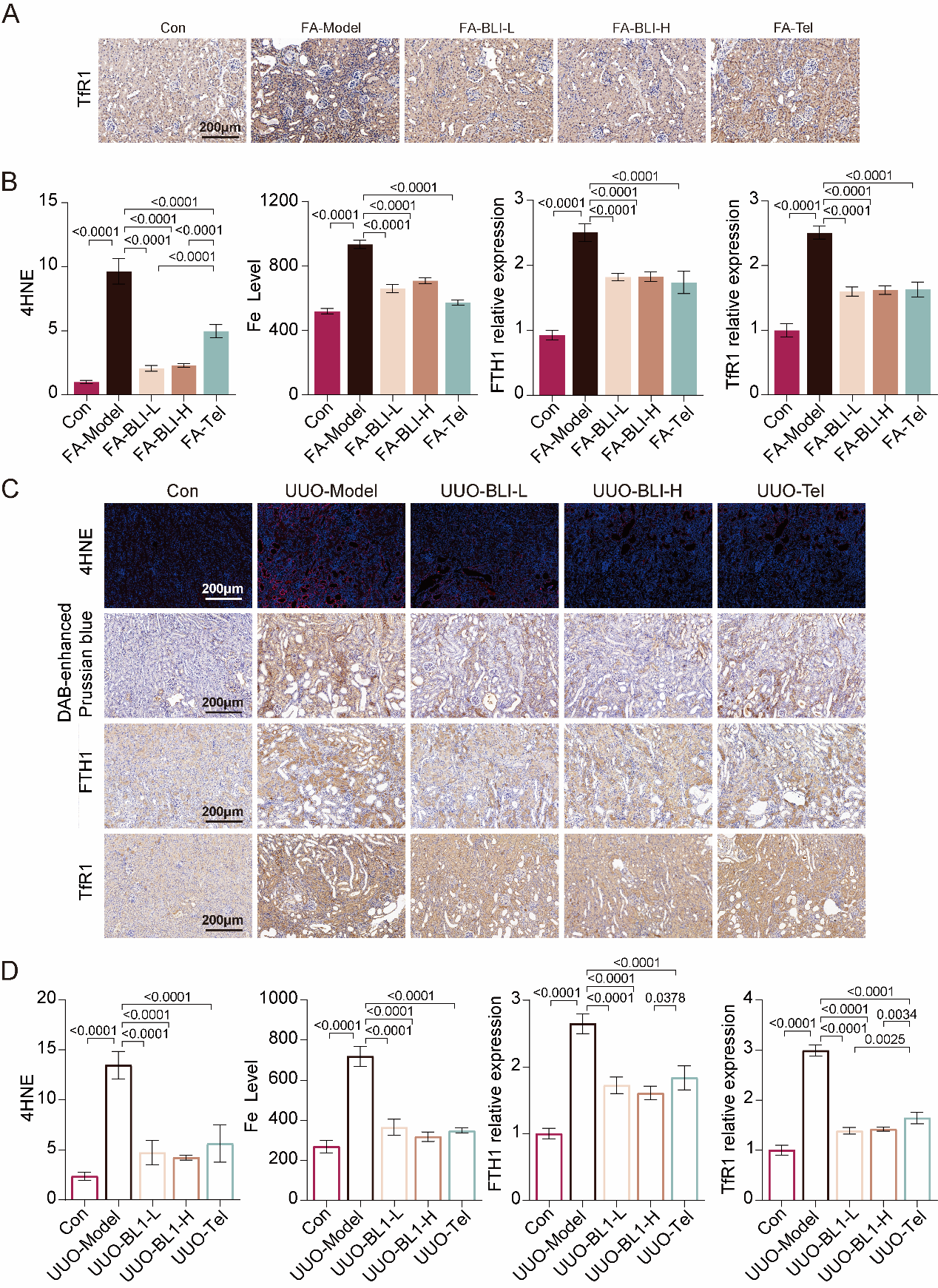


**Figure** **S5. BLI mitigates kidney ferroptosis in a mouse model.** (A) Results of immunohistochemical staining for TfR1 in the kidney (n=5). (B) Quantification of 4-HNE-, Fe^+2^-, and FTH1- or TfR1-positive regions in the kidney (n=5). (C) and (D) Representative images and quantitative results of 4-HNE or DAB-enhanced Prussian blue staining and immunohistochemical staining for FTH1 or TfR1 in the kidney (n=5). Significant P values are indicated on figure panels. Scale bars were as shown in the figure.


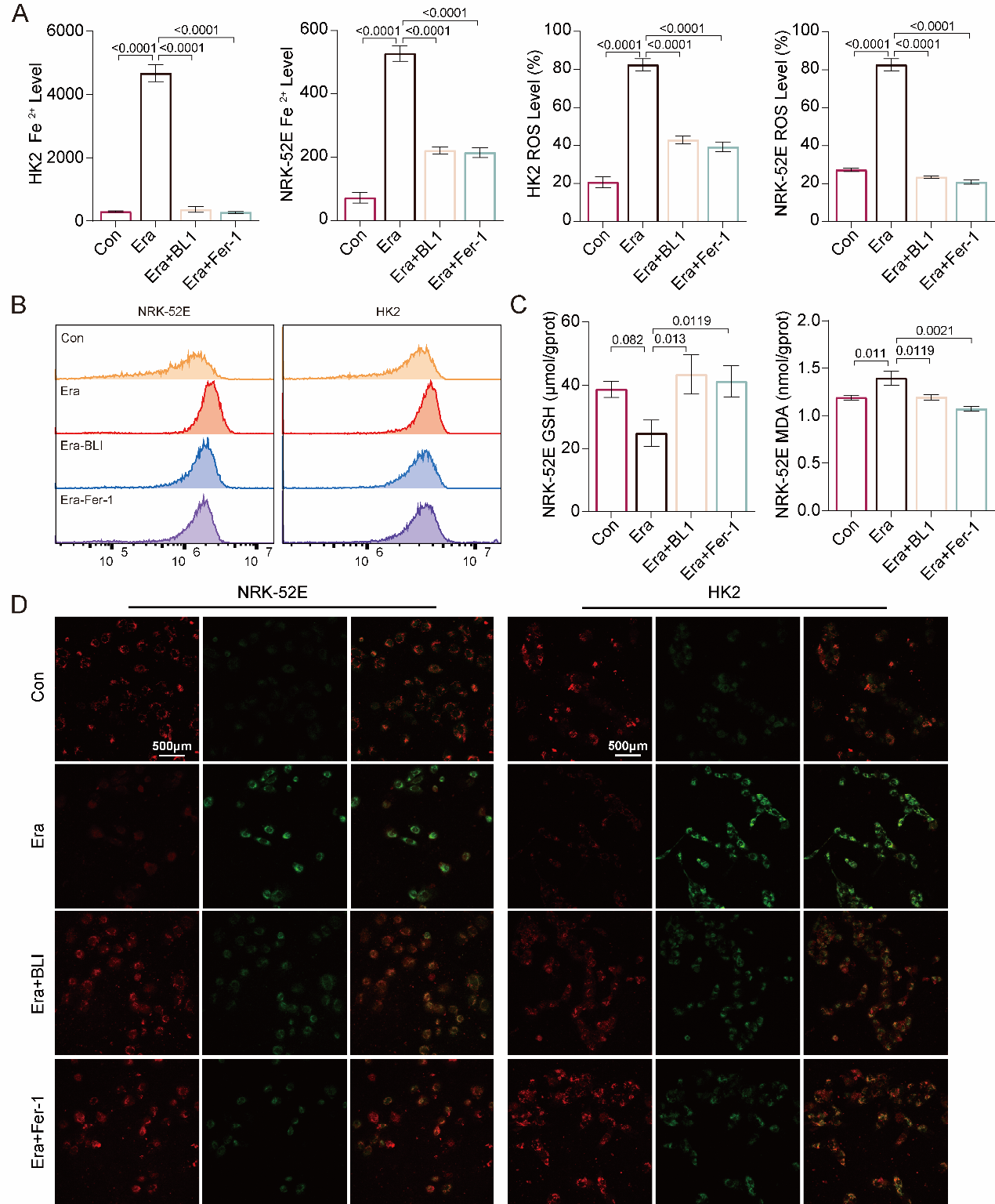
**Figure S6. BLI inhibits ferroptosis in HK2 and NRK-52E cells.** (A) Quantitation of Fe^+2^ and ROS in HK2 and NRK-52E cells. (B) The fluorescence intensity of ROS was analyzed by flow cytometry. (C) MDA and GSH levels in NRK-52E cells. (D) Representative images of JC-1-stained HK2 and NKR-52E cells in each group after treatment (n=3). Significant P values are indicated on figure panels. Scale bars were as shown in the figure.


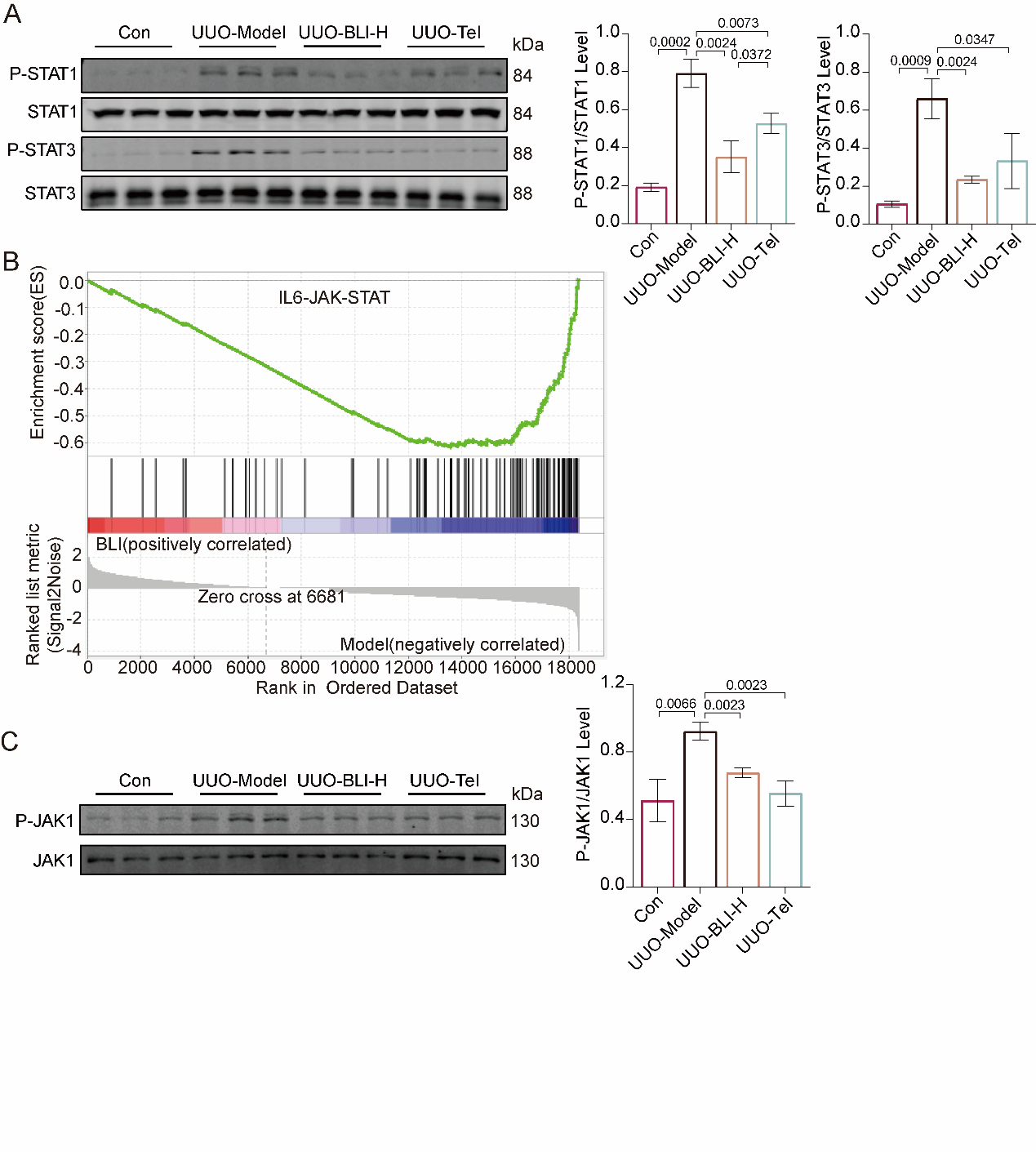


**Figure S7. BLI exerts a nephroprotective effect by targeting JAK1.** (A) Results of immunoblotting for p-STAT1 and p-STAT3 in the UUO-induced model (n=3). (B) Enrichment analysis of the GSEA of the kidney RNA-seq results of the FA+BLI group compared with the FA group. (C) Immunoblotting results of p-JAK1 in the UUO-induced model (n=3). Significant P values are indicated on figure panels.


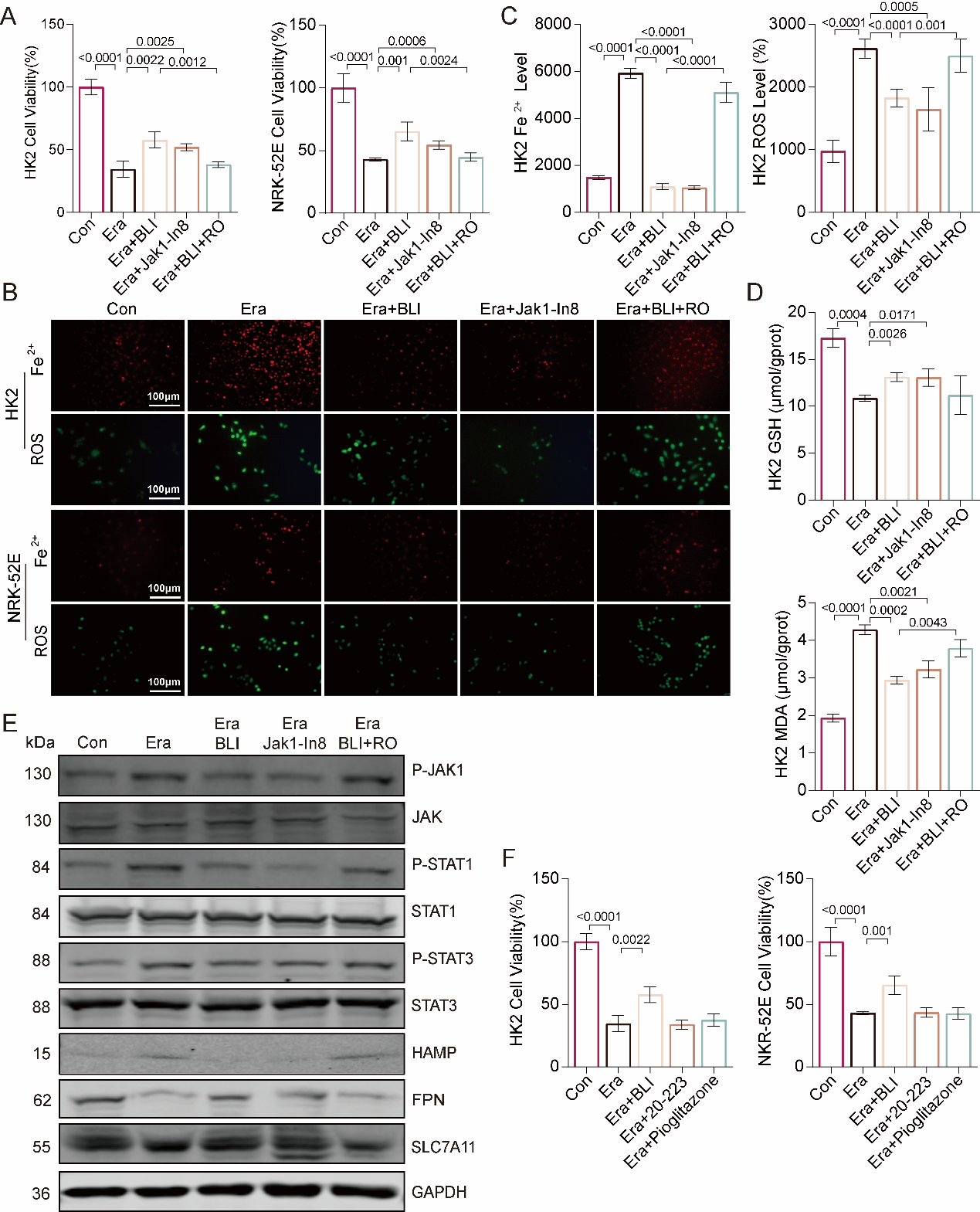


**Figure S8. BLI inhibits ferroptosis by inhibiting the JAK-STAT signaling pathway.** (A) Cell viability after treatment with drugs for 24 h (RO: RO8191, n=3). (A) and (C) Intracellular iron and ROS levels in cells (n=3). (D) MDA and GSH levels in cells (n=3). (E) Immunoblotting results (n=3). (F) Cell viability after treatment with drugs for 24 h (n=3). Significant P values are indicated on figure panels. Scale bars were as shown in the figure.


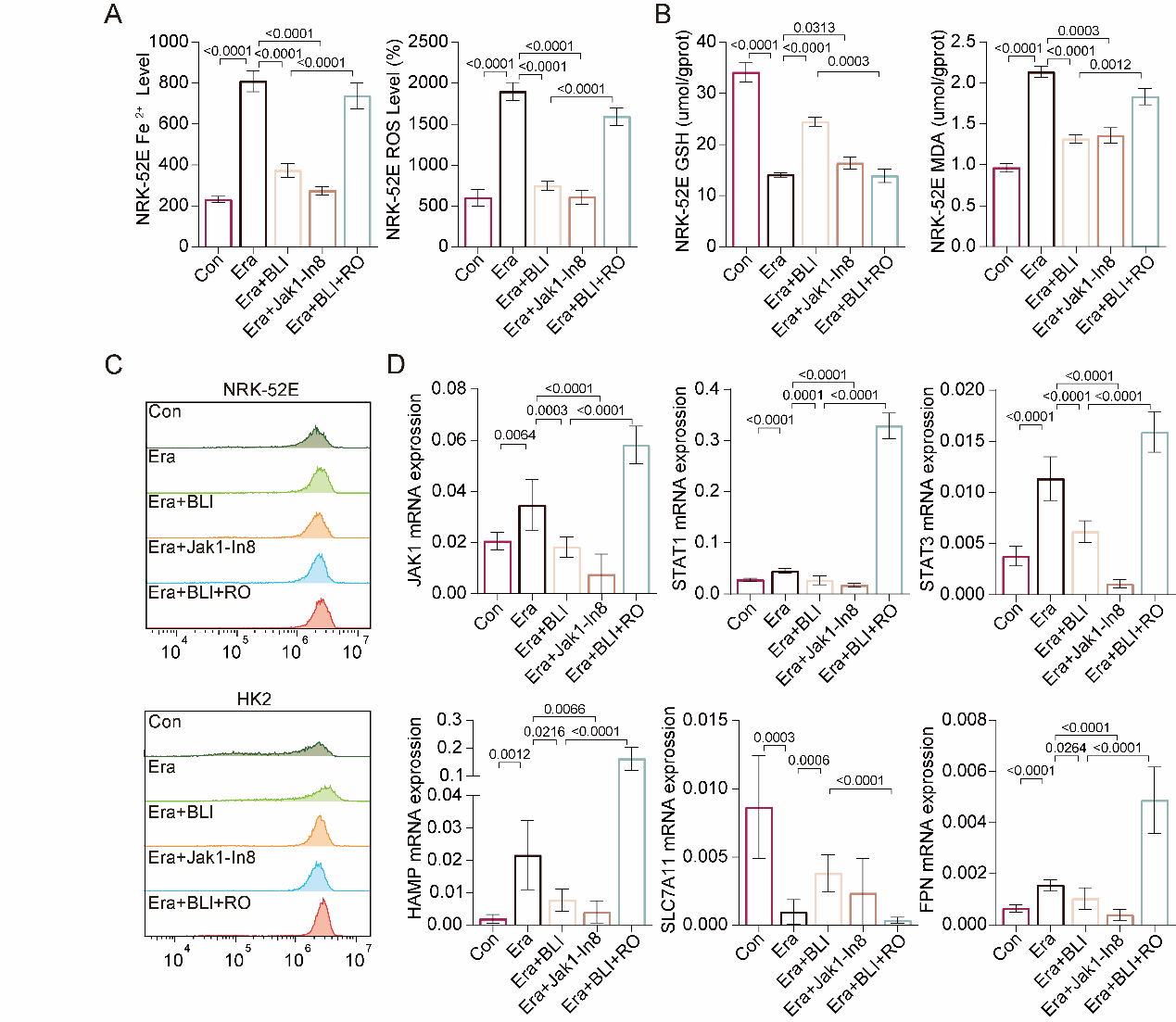


**Figure S9. BLI inhibits ferroptosis by inhibiting the JAK-STAT signaling pathway.** (A) Quantitation of Fe^+2^ and ROS in NRK-52E cells (n=3). (B) MDA and GSH levels in NRK-52E cells (n=3). (C) The fluorescence intensity of ROS was analyzed by flow cytometry (n=3). (D) RT-qPCR results of JAK1, STAT1, STAT3, HAMP, SLC7A11 and FPN in HK2 cells (n=3). Significant P values are indicated on figure panels.


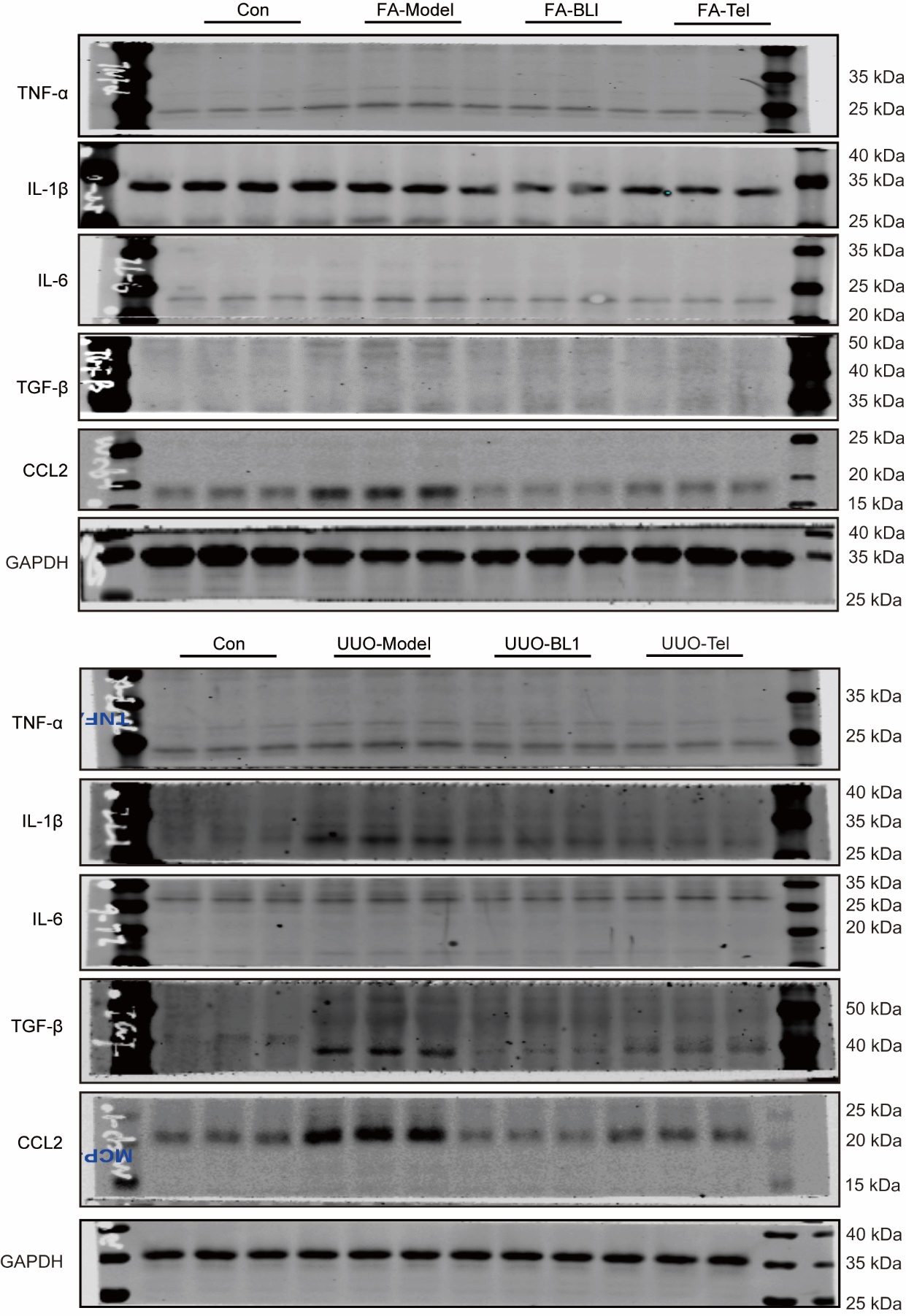


**Figure S10. Complete membrane images of all immunoblot images shown in Figure 2G.**


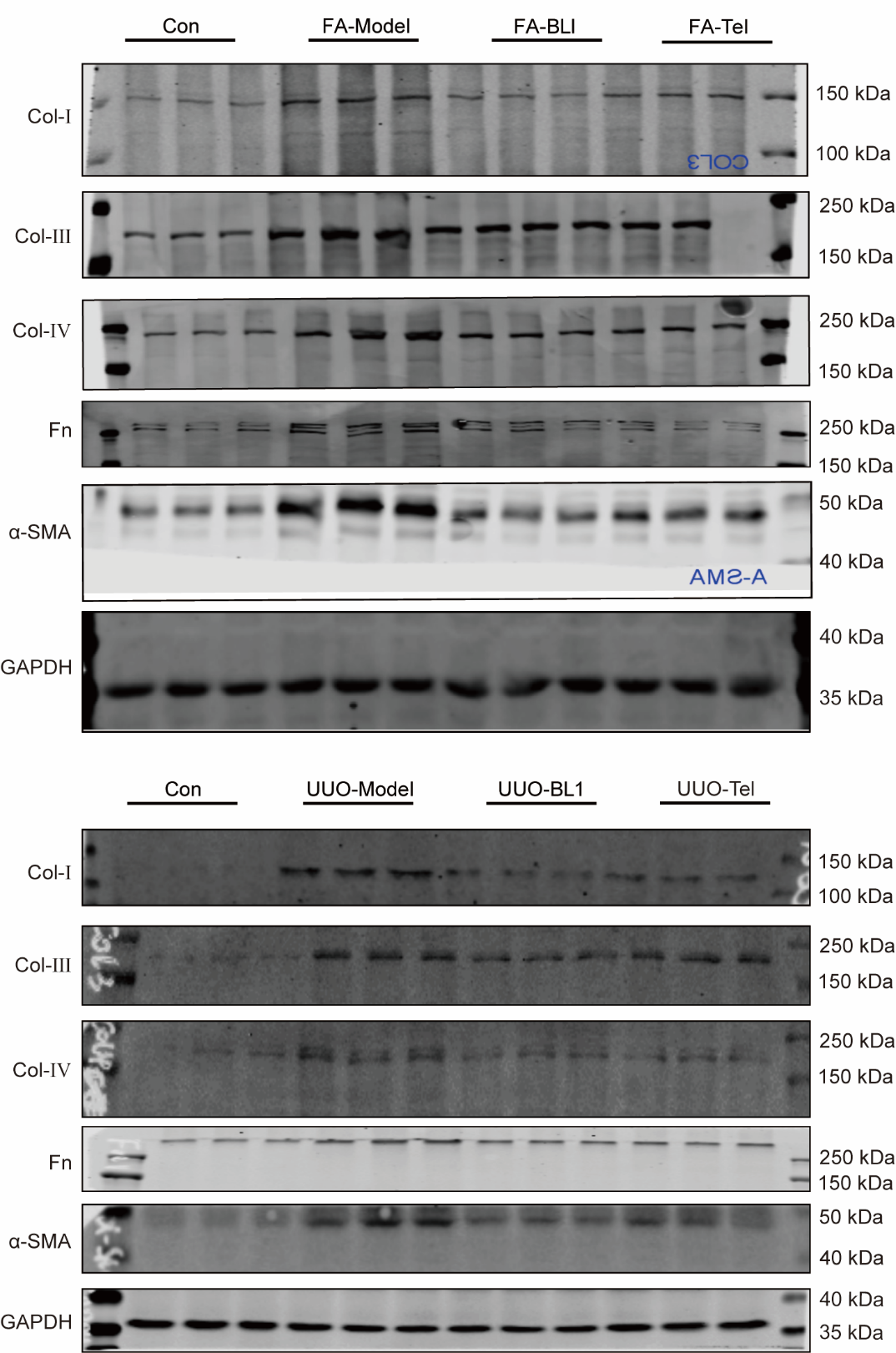


**Figure S11. Complete membrane images of all immunoblot images shown in Figure 3B, 3D.**


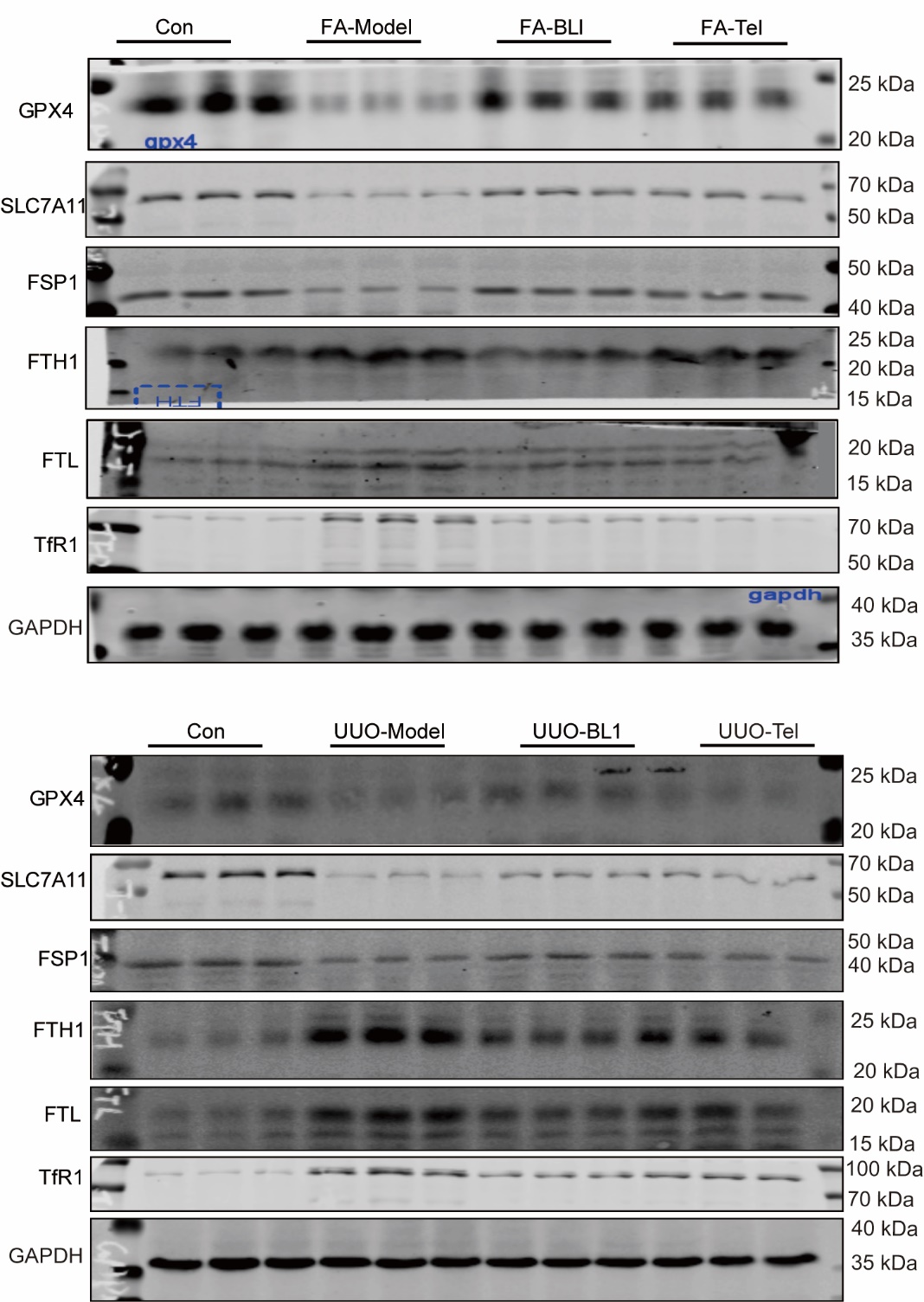


**Figure S12. Complete membrane images of all immunoblot images shown in Figure 4F**


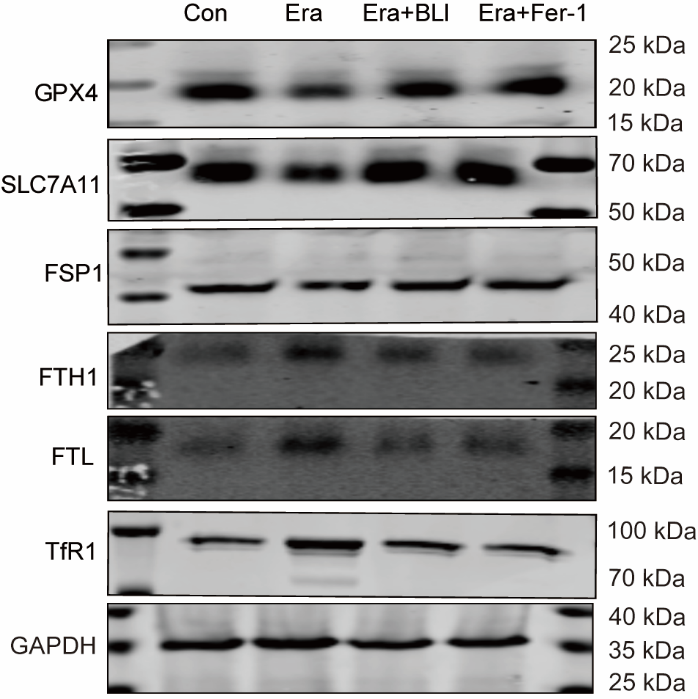


**Figure S13. Complete membrane images of all immunoblot images shown in Figure 5D.**


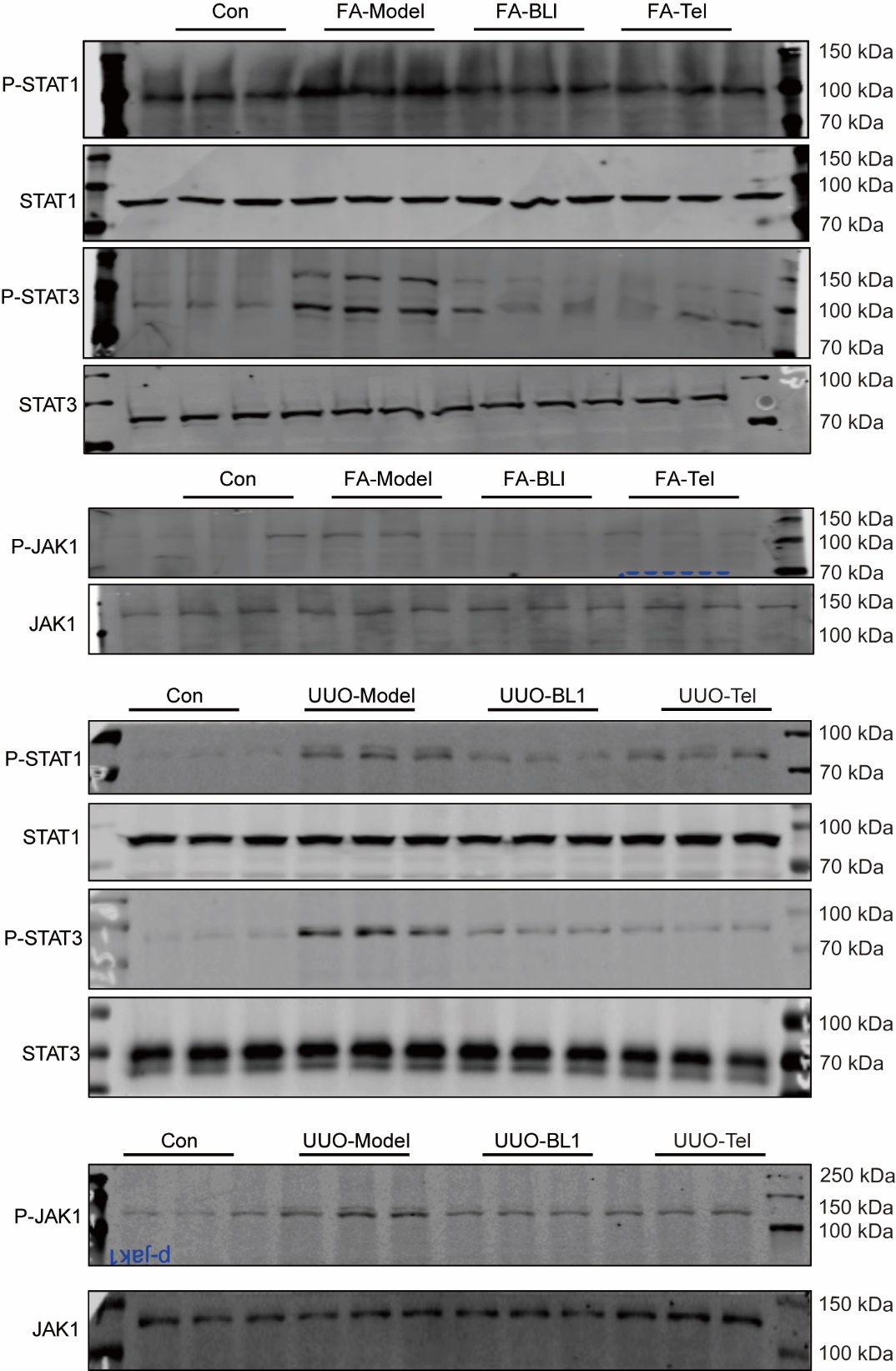


**Figure S14. Complete membrane images of all immunoblot images shown in Figure 6A, 6E, Figure S7A and Figure S7C**


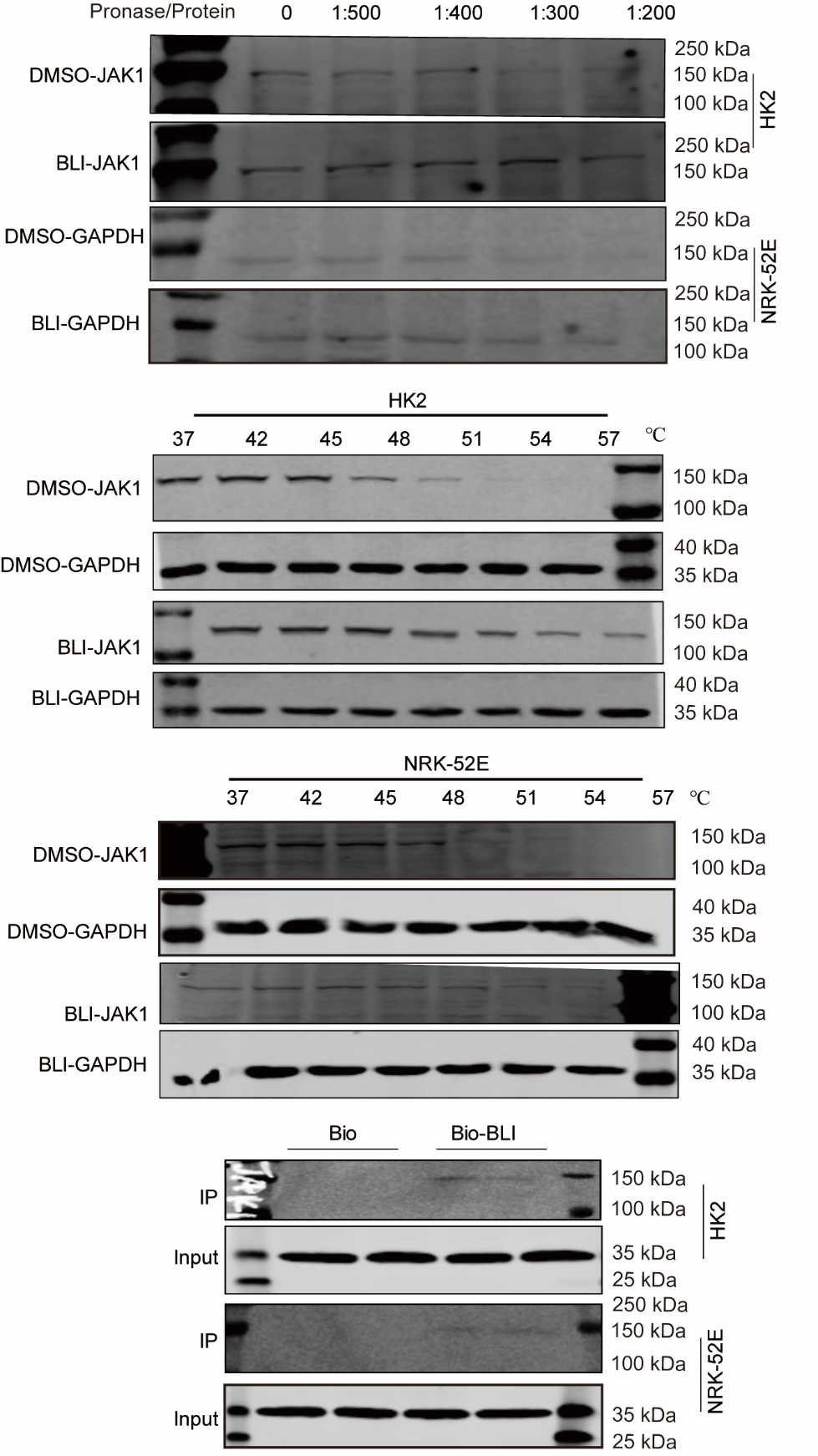


**Figure S15. Complete membrane images of all immunoblot images shown in Figure 6F, 6G, 6I, and Figure S8E.**


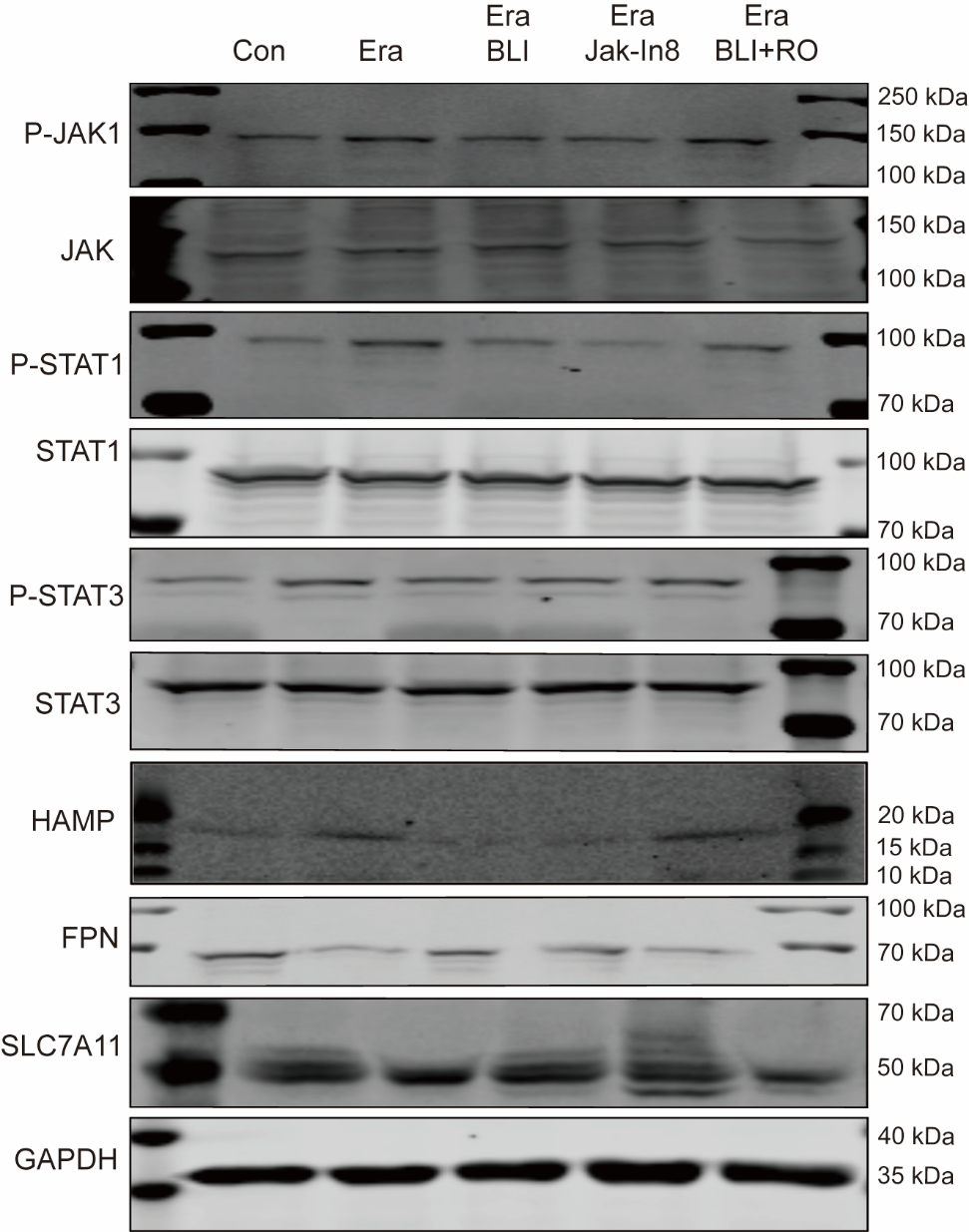


**Figure S16. Complete membrane images of the immunoblot images shown in Figure S8E.**

**Table S1. The gene sequences of mouse primers**

| Gene | Forward | Reverse |
| --- | --- | --- |
| *IL-1β* | TGGACCTTCCAGGATGAGGACA | GTTCATCTCGGAGCCTGTAGTG |
| *IL-6* | TACCACTTCACAAGTCGGAGGC | CTGCAAGTGCATCATCGTTGTTC |
| *TGF-β* | TGATACGCCTGAGTGGCTGTCT | CACAAGAGCAGTGAGCGCTGAA |
| *MCP-1* | GCTACAAGAGGATCACCAGCAG | GTCTGGACCCATTCCTTCTTGG |
| *TNF-α* | GGTGCCTATGTCTCAGCCTCTT | GCCATAGAACTGATGAGAGGGAG |
| *TfR1* | GAAGTCCAGTGTGGGAACAGGT | CAACCACTCAGTGGCACCAACA |
| *TfR2* | TCAGCGTGCTACACCTCAAAGC | CTCAATGAGGCTGACGAGAAGG |
| *DMT1* | TTGCAGCGAGACTTGGAGTGGT | GCTGAGCCAATGACTTCCTGCA |
| *FTH1* | GCCGAGAAACTGATGAAGCTGC | GCACACTCCATTGCATTCAGCC |
| *HAMP* | CAGCACCACCTATCTCCATCAAC | AAGTGGGTGTCTCGCCTCCTTC |
| *FTL* | CCTCGAGTTTCAGAACGATCGC | CCTGATTCAGGTTCTTCTCCATG |
| *NCOA4* | TGCCATTGGTCTTCAGGCTCCT | CAGGCATCGCTGAAGAAACTGC |
| *ACSL4* | CCTTTGGCTCATGTGCTGGAAC | GCCATAAGTGTGGGTTTCAGTAC |
| *SLC7A11* | CTTTGTTGCCCTCTCCTGCTTC | CAGAGGAGTGTGCTTGTGGACA |
| *DHODH* | TGAGGAGCCTACAGGGAAAGAC | ACGCTGGCAATGTCCTCCTTGT |
| *GPX4* | CCTCTGCTGCAAGAGCCTCCC | CTTATCCAGGCAGACCATGTGC |
| *FSP1* | AGCTCAAGGAGCTACTGACCAG | GCTGTCCAAGTTGCTCATCACC |
| *FPN* | CCATAGTCTCTGTCAGCCTGCT | CTTGCAGCAACTGTGTCACCGT |
| *KIM-1* | CTGGAATGGCACTGTGACATCC | GCAGATGCCAACATAGAAGCCC |
| *Ngal* | ATGTCACCTCCATCCTGGTCAG | GCCACTTGCACATTGTAGCTCTG |

**Table S2. The gene sequences of human primers**

| Gene | Forward | Reverse |
| --- | --- | --- |
| JAK1 | CTGTCTACTCCATGAGCCAGCT | CCTCATCCTTGTAGTCCAGCAG |
| *STAT1* | ATGGCAGTCTGGCGGCTGAATT | CCAAACCAGGCTGGCACAATTG |
| *STAT3* | CTTTGAGACCGAGGTGTATCACC | AGGAGTCTAACAACGGCAGCCT |
| *HAMP* | CTGACCAGTGGCTCTGTTTTCC | AAGTGGGTGTCTCGCCTCCTTC |
| *SLC7A11* | TCCTGCTTTGGCTCCATGAACG | AGAGGAGTGTGCTTGCGGACAT |
| *FPN* | GAGACAAGTCCTGAATCTGTGCC | TTCTTGCAGCAACTGTGTCACAG |
| *GPX4* | ACAAGAACGGCTGCGTGGTGAA | GCCACACACTTGTGGAGCTAGA |
| *FSP1* | GACTCCTTCCACCACAATGTGG | CAGCACCATCTGGTTCTTCAGG |
| *FTH1* | TGAAGCTGCAGAACCAACGAGG | GCACACTCCATTGCATTCAGCC |
| *FTL* | GAGACAGGTCTCCCACAAACAC | GTGGTAAGGACATCGCTTTTCCG |
| *TfR1* | GCACCTCAAAGCCGTAGTGTAC | CCACCTGTTCATAGAGAGTCTGC |
